# Laminar compartmentalization of attention modulation in area V4 aligns with the demands of visual processing hierarchy in the cortex

**DOI:** 10.1038/s41598-023-46722-8

**Published:** 2023-11-09

**Authors:** Xiang Wang, Anirvan S. Nandy, Monika P. Jadi

**Affiliations:** 1 Interdepartmental Neuroscience Program, Yale University, New Haven, CT 06511 USA; 2 Department of Psychiatry, Yale University, New Haven, CT 06511 USA; 3 Department of Neuroscience, Yale University, New Haven, CT 06511 USA

**Keywords:** Cognitive neuroscience, Attention, Neuroscience, Extrastriate cortex

## Abstract

Attention selectively enhances neural responses to low contrast stimuli in visual area V4, a critical hub that sends projections both up and down the visual hierarchy. Veridical encoding of contrast information is a key computation in early visual areas, while later stages encoding higher level features benefit from improved sensitivity to low contrast. How area V4 meets these distinct information processing demands in the attentive state is unknown. We found that attentional modulation in V4 is cortical layer and cell-class specific. Putative excitatory neurons in the superficial layers show enhanced boosting of low contrast information, while those of deep layers exhibit contrast-independent scaling. Computational modeling suggested the extent of spatial integration of inhibitory neurons as the mechanism behind such laminar differences. Considering that superficial neurons are known to project to higher areas and deep layers to early visual areas, our findings suggest that the interactions between attention and contrast in V4 are compartmentalized, in alignment with the demands of the visual processing hierarchy.

## Introduction

Voluntary attention is essential for sensory guided behavior and memory formation^1^. Failures in sensory processing and selective attention are aspects of many mental illnesses, including schizophrenia and mood disorders^2–4^. Visual spatial attention plays a critical role in visual sensory processing: It allows improved perception of behaviorally relevant target stimuli among competing distractors by boosting the apparent visibility of the target^5^. At the neuronal level, attention modulates the activity of cortical neurons that encode an attended visual stimulus at various stages of visual processing^6–12^. In visual areas such as V4 and MT, attention modulates neuronal mean firing rates, increases their firing reliability, and reduces the co-variability among pairs of neurons^11,13–16^. However, the computational principles that underlie the activity of neuronal populations that represent both sensory information and the attentional state remain poorly understood^17^.

Object recognition is mediated by a hierarchy of cortical visual processing areas that form the ventral visual stream. Contrast is a key feature of the visual scene that aids object recognition, and the encoding of contrast information is arguably one of the most important computations performed by early visual areas. On the other hand, visual features represented in higher areas such as the inferotemporal (IT) cortex benefit from improved sensitivity to low contrast stimuli^18,19^. Visual area V4 is a critical hub in the ventral stream that sends feedforward projections to areas such as IT and feedback projections to early visual areas^20–22^. Attention has been shown to selectively enhance the responses to low contrast stimuli^23,24^. Attention-mediated selective enhancement of low contrast features could potentially reinforce invariant representations in higher object recognition areas downstream of V4^25^. However, such a bias in the attention-modulated feedback from V4 to upstream visual areas is inconsistent with the contrast- independent attentional modulation observed in these areas^26,27^.

One possibility is that distinct subpopulations in V4 target upstream and downstream areas, and display divergent attention effects. Indeed, the sensory cortical sheet, including area V4, is not a homogeneous piece of tissue along its depth; rather, it has a six-layered or laminar structure made up of multiple cell classes, of both excitatory and local inhibitory kind, with largely stereotypical anatomical connectivity between and within layers^28^. Layer 4 (the *input* layer) of visual cortex is considered as the primary target of projections carrying visual information from early areas^29,30^. But some reports suggest layers 5/6 as an additional target^31–33^. Visual information is then processed by local neural subpopulations as it is sent to layers 2/3 (the *superficial* or *supragranular* layers) and layers 5/6 (the *deep* or *infragranular* layers), which serve as output nodes in the laminar circuit^34,35^. The superficial layers feed information forward principally to downstream visual areas^36,37^, whereas the deep layers send feedback information mainly to upstream early visual areas^30,38–41^. This anatomical organization raises the possibility of distinct functional roles^42^ and differential attentional modulation of sensory representation among cell-class and layer-specific neural subpopulations. In support of this idea, a recent study of simultaneous depth recordings in visual area V4 has shown layer-specific attentional modulation of average neuronal responses, reliability of responses, and correlations between responses of pairs of neurons^43^. Understanding the modulation of sensory representation in laminar subpopulations is thus crucial to elucidate the fundamental principles of attention-dependent sensory computations in the neocortex. In the context of a spatial attention-demanding task, we hypothesized that the attentional modulation of contrast computations in area V4 is not homogeneous, but rather is layer- and cell-class specific and that these differences reflect different computational demands on these subpopulations: feedforward signals to facilitate invariant visual representations in higher visual areas and feedback signals to support the contrast-independent attention modulation observed in early visual areas. Considering their key contribution to feedback projections to early visual areas, we expect that projection neurons in the deep layers show uniform attentional modulation across all contrasts in order to minimally impact the faithful representation of the contrast landscape in their target areas.

In this study, we characterized layer- and cell-class specific neural subpopulations from extracellular recordings of single neurons within area V4 of macaque monkeys performing an attention-demanding orientation change detection task. Using unsupervised clustering techniques on waveform durations without a priori assumptions about the underlying distribution, we distinguished three functional clusters of neurons. We distinguished layer identities—superficial, input or deep—of these neurons using features of local field potentials. To test our hypothesis, we characterized the attentional modulation of contrast response functions in these sub-populations. We interpreted our findings within a computational framework of attentional modulation of contrast responses^44^, which yielded predictions for distinct mechanistic roles of these neural subpopulations in attentive perception.

## Results

**Figure 1:**
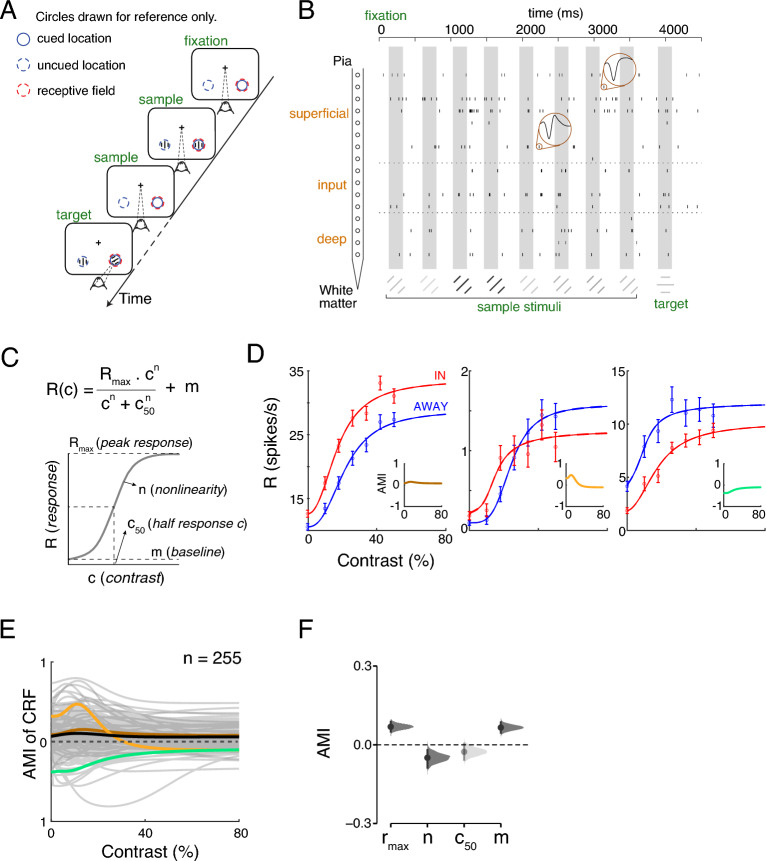
Attentional modulation of Contrast Response Function. (**A**) Orientation change detection task. While the monkey maintained fixation, two oriented Gabor stimuli were flashed on and off at two locations: one within the RF overlap region of the recorded V4 column and the other at a location of equal eccentricity across the vertical meridian. The covert attention of the monkey was cued to one of the two locations. One of the two stimuli changed its orientation at an unpredictable time. The monkey was rewarded for making a saccade to the location of orientation change (95% probability of change at the cued location; 5% probability at uncued location [foil trials]). If no change happened (catch trials), the monkey was rewarded for maintaining fixation. (**B**) An example trial showing single-unit activity across the cortical depth in the attend-in condition. The time axis is referenced to the appearance of the fixation spot. Spikes (vertical ticks) in each recording channel come from the single unit with the highest spike rate in this trial. The gray boxes depict stimulus presentation epochs. In this particular trial, 8 sample stimuli with different contrasts were presented, followed by a target stimulus flash with an orientation change that the monkey responded to correctly. Spike waveforms for two single units are shown. (**C**) The mathematical function we used to fit neuronal contrast response functions is shown on the top and the interpretation of each parameter is shown below. (**D**) The best-fitting contrast response functions of three example neurons in “attend in” and “attend away” conditions. Mean ± SEM. Insets show the attentional modulation indices calculated as a function of contrast. (**E**) The attention modulation index (AMI) as a function of contrast for each of the 255 visually responsive single units. Highlighted are the three example units in (**C**) and the mean over the whole population (black). (**F**) The bootstrap sampling distribution of mean AMI for each CRF parameter. The average is depicted as a dot. Each 95% confidence interval (CI) is indicated by the ends of the vertical error bars. The faded color represents that the 95% CI include 0.

In the primate visual system, cortical sensitivity to features such as luminance contrast varies with the locus of spatial attention; contrast response functions (CRF) of cortical neurons are measured to quantify this dependence^16,24,45^. However, the laminar- and cell-class specific dependence of the CRF on attentive state is not known. Using linear array electrodes or single tungsten electrodes, we recorded neuronal activity from well-isolated single units (*n* = 337 for laminar recordings, *n* = 73 for single-electrode recordings) in visual area V4 of two rhesus macaques (right hemisphere in monkey A, left hemisphere in monkey C) during an attention demanding orientation change detection task (Fig. 1A,B; see Methods and *SI Text*). For laminar recordings, we used current source density (CSD) analysis to identify different laminar compartments (*superficial*, *input*, and *deep*), and assigned isolated single units to one of the three layers (see *SI Text*). The receptive fields along the cortical depth were well aligned, as shown by a previous study using the same dataset^43^, suggesting the recordings were from the same column. In the main experiment, we presented a sequence of paired Gabor stimuli with different contrasts (Fig. 1B); one stimulus was presented inside the receptive fields (RFs) of the recorded neurons and the other at an equally eccentric location across the vertical meridian. Attention was cued either to the stimuli within the neurons’ RFs (“attend-in”) or to the stimuli in the contralateral visual hemifield (“attend-away”). One of the two stimuli changed its orientation (95% valid cue) at a time unknown to the animal, and the monkey was rewarded for detecting the orientation change by making a saccade to the corresponding location.

### Attentional modulation of contrast response function

To examine the effects of attention on individual neurons, we used the method of ordinary least squares to fit a hyperbolic ratio function to each neuron’s contrast responses, separately for each attention condition (Fig. 1C,D, S1A,B). This function is described by four parameters: $R_{max}$, $c_{50}$, $m$, and $n$, where $R_{max}$ is the attainable maximum response, $c_{50}$ is the contrast at which neuronal response is half-maximal, $m$ is the baseline activity, and $n$ describes the nonlinearity of the function (Fig. 1C). Attention effects differ considerably for individual neurons. Attention either enhances or suppresses neuronal responses at different contrast levels (Fig. 1D). We quantified the effect of attention on every recorded neuron by computing the attentional modulation index (AMI) using contrast response functions (CRFs) from both attention conditions (see *SI Text*). We saw a significant variance of AMI values at each contrast level (Fig. 1E). We also examined how attention impacts the values of best-fitting CRF parameters (Fig. 1F, S1C). The mean AMIs for $r_{max}$ and $m$ are significantly higher than zero (Wilcoxon signed rank test, *p* < 0.001 for both distributions, Fig. S1C), which is consistent with previous observations in V4^46^. The same percentage change in $r_{max}$ and $m$ (15% increase) supports an effect of contrast independent scaling by attention. The average modulations of $c_{50}$ and $n$ are statistically smaller than zero (Wilcoxon signed rank test, *p* < 0.05), suggesting an increased sensitivity to low contrast stimuli and a reduction in the sensitivity to contrast change, respectively.

In order to achieve a deeper understanding of the data distributions, we also analyzed the AMIs for CRF parameters as well as other important quantities within the framework of Estimation Statistics (see *SI Text*). This method has been widely adopted in scientific disciplines and is becoming a new standard in some neuroscience journals^47^. It generates a richer representation of the data than the more commonly used Null-Hypothesis Significance Testing (NHST). As advocated by Calin-Jageman and Cumming^48^, the Estimation Statistics approach is complementary to the NHST approach and offer a principled way to measure effect sizes coupled with estimates of uncertainty, yielding interval estimates of effect size. The Estimation Statistics of the mean difference from 0 support the statistical significance of attention effects on $r_{max}$, $n$, and $m$ (Fig. 1F). Moreover, the modulations of CRF parameters are variational across subjects (Fig. S5A,B). These results indicate that the overall effect of attention on V4 neuron responses cannot be simply explained as a response gain or a selective boosting of low contrast. Modulations in parameters of the contrast response function are highly variable across neurons.

### Classification of single units using electrophysiological features

**Figure 2:**
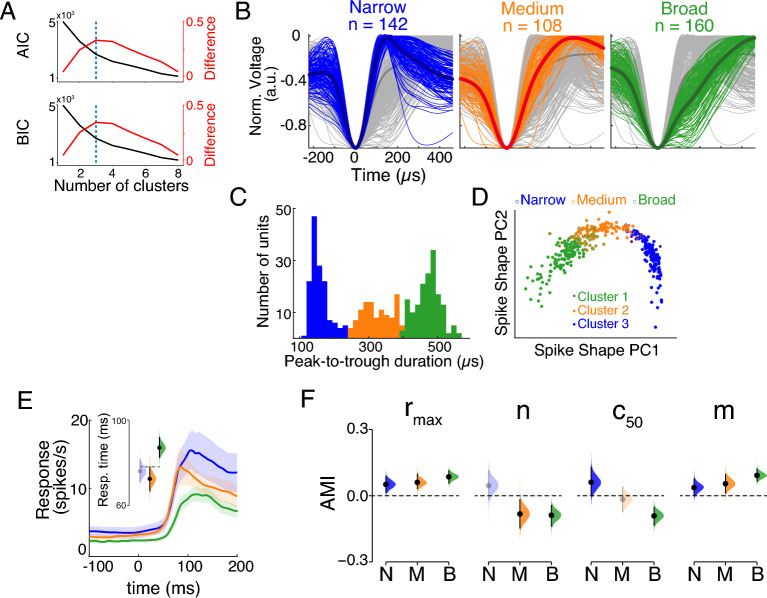
Classification of Single Units Using Waveform Widths. (**A**) Information metrics for different number of clusters. AIC: Akaike information criterion; BIC: Bayesian information criterion. The elbow point (marked by purple dotted line) was detected by the kneedle algorithm as the local maxima of the difference curve (red) computed from the normalized AIC or BIC values. (**B**) Mean waveforms for all 410 single units colored by their cluster identities. Waveforms were smoothed using spline interpolation and their heights were normalized to help compare spike widths. The mean waveform averaged across all units was shown in dark gray and the mean waveforms for each cluster were shown in dark colors. (**C**) Distribution of action potential waveform width (peak-to-trough duration) for all 3 clusters collapsed across layers. The distribution is clearly multimodal (Hartigan’s dip test, *p* < 0.001). (**D**) In the principal component (PC) space of spike shape, single units were colored either based on their spike width classifications (open circles; Narrow, Medium, Broad) or by running the *k*-means clustering algorithm with the first 2 PCs (closed circles). The clusters generated from the peak-to-trough duration match the ones classified by spike shape PCs, suggesting that peak-to-trough duration is an efficient measure to capture the variance of spike shape data. (**E**) Lines and shading show mean ± SEM firing rate estimates with variable kernel bandwidths for 3 classes around the stimulus onset (Narrow, *n* = 43; Medium, *n* = 54; Broad, *n* = 78 neurons). The inset shows the bootstrap sampling distribution of the mean response time for each cluster. Response time is the interval between the stimulus onset and the time when the post-stimulus firing rate is consistently greater than the 95% bootstrap CI of the pre-stimulus mean firing rate. The dashed line in the inset indicates the upper bound of the 95% CI of the Medium class. Faded color of Narrow indicates the overlap of its CI with Medium’s. The difference between Medium and Broad was substantiated by the Wilcoxon rank sum test (Bonferroni corrected, *n* = 3, p_medium⇔broad_ < 0.001). See Table S2 in *SI Text* for ANOVA tests. (F) Bootstrap sampling distributions of AMIs of CRF parameters for each cell class. Distributions with CIs including 0 are displayed in faded colors. All visually responsive single units were included (Narrow, *n* = 71; Medium, *n* = 75; Broad, *n* = 109). The asterisk indicates that the median of the distribution is significantly different from 0 (Wilcoxon signed rank test, *p* < 0.05). For the swam plot of AMIs, see Fig. S2E.

To investigate whether attention modulates different functional classes of neurons uniformly or differentially, we characterized classes of single units based on the peak-to-trough duration (PTD). Properties of the action potential waveform, especially the PTD, have been extensively used to classify neurons into narrow- (putative inhibitory) and broad-spiking (putative excitatory) cells^14,49–54^. Given the insufficient and biased sampling of single units within each monkey (Fig. S5C,D), we decided to apply the classification on the data aggregated across both animals. The shapes of average spiking waveform for all single units in our data were also highly variable (Fig. 2B), and the distribution of PTD was clearly multimodal (Fig. 2C, Hartigan’s dip test, *p* < 0.001)^55^. We exploited the information structure in the entire waveforms by applying principal component analysis (PCA). The correlation pattern between the first two components of the PCA (cumulative percentages of explained variance: 59.62%, 83.10%) supported the idea that neurons can be separated into meaningful clusters by waveform shape measures (Fig. 2D).

We used a meta-clustering analysis based on the *k*-means clustering algorithm (see *SI Text*) to identify clusters of isolated single units^56,57^. To select the optimal number of clusters (*k*), we computed Akaike information criterion (AIC) and Bayesian information criterion (BIC) for each *k*. Elbow points of the two criteria (see *SI Text*) supported the selection of the 3-cluster solution (Fig. 2A). Narrow-spiking cells become a cluster by themselves, while a medium-spiking cluster emerged from those previously classified as broad-spiking cells (Fig. 2B)^14,43^. We also derived the same clusters from the laminar recordings alone that constitute 82% of the data, supporting the robustness of the trimodal distribution of waveform widths (Fig. S6).

One assumption we made to use the PTD as a clustering feature was that it captures a significant amount of the variation of neurons’ spiking waveforms. We tested this assumption by clustering neurons in the principal component space of the action potential waveform and comparing them with neuronal groups classified by their PTD. We found that the three clusters separated by waveform principal components were highly consistent with those classified by the PTD (82.4% of units with the same cluster identity) (Fig. 2D).

The clusters differ in terms of their firing rate profiles across layers (Fig. 2E, S2A). Although not statistically significant, narrow-spiking neurons exhibited higher firing rates than medium- and broad-spiking clusters when averaged across layers (mean 10.1 Hz compared to 8.9 Hz and 6.5 Hz). It is in agreement with previous findings that narrow-spiking single units, considered putative inhibitory interneurons, show higher firing rates than units with broader PTD, thought to be putative excitatory cells^14,58–61^.

Neuronal subclasses in the cortex are differentially targeted by feedforward inputs, which gives them functional distinction^33,62,63^. Subclasses that are strongly driven by feedforward inputs are expected to exhibit shorter latencies in their response to feedforward stimulation. To investigate the distinct ways in which our identified clusters participate in the laminar cortical circuit, we next examined the response latency of these neuronal classes to visual stimuli (see *SI Text*). For each single unit, we estimated its firing rate using a variable Gaussian kernel approach (bandwidth optimized for every time bin)^64^ and defined the response time as the post-stimulus delay at which its firing rate surpassed the upper bound of the 95% confidence interval (CI) calculated from its pre-stimulus spontaneous activity. We then averaged the response time across units within a cluster (Fig. 2E) and within a layer (Fig. S2B). When averaged across layers, medium-spiking cells showed significantly shorter response time than the other two classes (Fig. 2E). We observed a similar pattern in individual subjects (Fig. S5E,F). A layer specific analysis revealed that medium-spiking neurons robustly exhibited faster response than broad-spiking cells in the deep layers (Fig. S2B). A similar trend exists in the superficial and input layer, but was not statistically significant. To verify that such latency differences between Medium and Broad classes were not specific to the method applied, we also estimated single units’ firing rates with fixed kernel bandwidths (selected from 1 to 10 ms) and defined the response time as the first of five consecutive time bins after stimulus onset that reach a firing rate higher than the maximum of the pre-stimulus period^50^. The difference between medium- and broad-spiking neurons still existed when averaged across layers (Fig. S2C). Moreover, it is possible that the Narrow or Broad cluster contain multiple different cell types that prolong their average latencies. To rule out this possibility, we examined the standard deviation of the waveforms for every cluster, and we found that the Narrow and Broad classes did not possess the most variable waveforms (Fig. S2D), which suggests that the short response time of Medium class was not caused by a more diverse composition of cell types in the other two clusters. These results provide further support that medium-spiking neurons represent a subpopulation that is functionally distinct from the broad-spiking cells. Their shorter latencies imply that the medium-spiking subpopulation might be the first group of cells that receive visual information in the input and deep layers.

### Cell-class and layer-specific attentional modulation

We next examined how attention modulates contrast responses for each cell class. We first checked the average best-fitting CRFs for every cluster under different attention conditions (Fig. S2F). We observed that all clusters exhibited robust contrast sensitivity across different layers and different attention conditions. We further computed the AMIs of best-fitting CRF parameters for every cell class. The pattern of modulations of CRF parameters was distinct for individual cell classes (Fig. 2F). All three clusters showed significant positive modulations of $r_{max}$ and $m$. Medium and Broad clusters are negatively modulated in their nonlinearity parameter $n$, an effect that enhances low-contrast responses and suppresses high-contrast responses (reduced overall contrast sensitivity). Notably, narrow-spiking and broad-spiking neurons exhibited changes of $c_{50}$ by attention in opposite directions, resulting in a non-significant modulation of $c_{50}$ of unclassified neurons. Although cell classes exhibited inconsistent attention effects across animals, presumably due to the biased sampling of units within each subject, the *differences* among clusters still exist (Fig. S5G,H).

**Figure 3:**
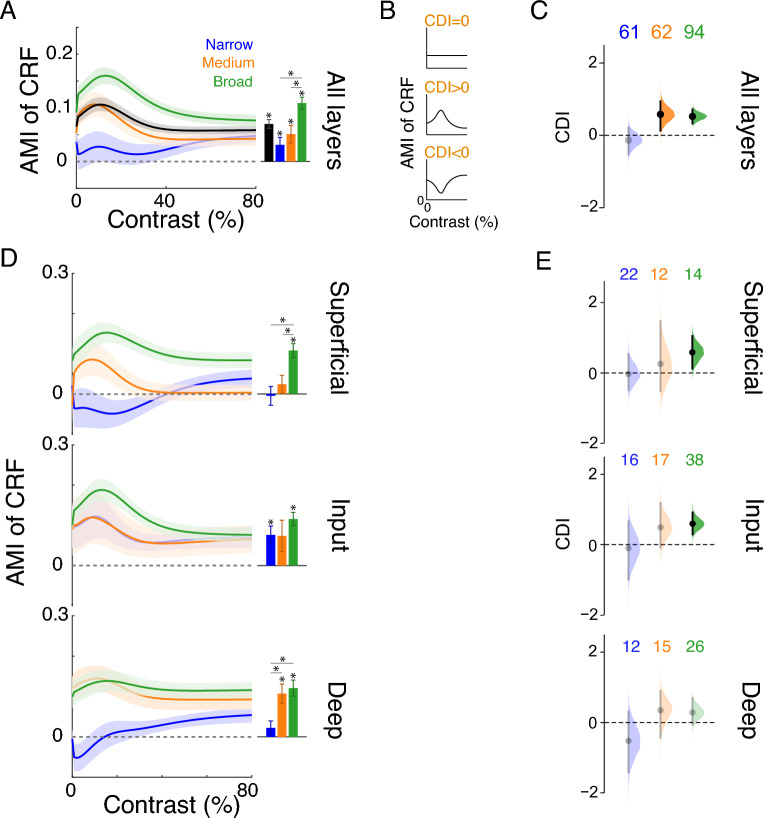
Contrast Dependency of AMI is Cell-Class and Layer-Specific. See Table S2 in SI Text for ANOVA tests of (**A**,**C**,**D**,**E**). (**A**) *Left*: the AMI as a function of contrast averaged across single units in each cluster. Mean ± SEM. The population mean is shown in black. *Right*: the mean AMI averaged across contrasts for each cluster or for all neurons. Asterisk indicates either the distribution is significantly different from zero (Wilcoxon signed rank test, *p* < 0.05) or two distributions are significantly different (Wilcoxon rank sum test, Bonferroni corrected, *n* = 3, *p* < 0.0167). For AMI curves of individual neurons in each cluster, see Fig. S3A. (**B**) The schematic showing the interpretation of different signs of CDI in terms of the AMI. The sign of CDI indicates the pattern of attentional modulation of contrast response. (**C**) The bootstrap sampling distribution of the mean CDI for each cell class combined across layers. The number of units excluding outliers for each cell class is shown on the top. Distributions with CIs inclusive of 0 are shown in faded colors. Clusters with significantly positive mean CDI were substantiated by the Wilcoxon signed rank test (*p* < 0.01). (**D**) Layer-wise AMI (mean ± SEM) of contrast responses for each cell class as a function of contrast (*left*) or averaged across contrasts (*right*). Asterisk indicates either the distribution is significantly different from zero (Wilcoxon signed rank test, *p* < 0.05) or two distributions are significantly different (Wilcoxon rank sum test, Bonferroni corrected, *n* = 3, *p* < 0.0167). (**E**) Layer-wise bootstrap sampling distribution of the mean CDI for each cell class. Distributions with CIs inclusive of 0 are illustrated in faded colors. Positive mean CDIs for Broad in superficial and input layers were supported by the Wilcoxon signed rank test (*p* < 0.05). For the swarm plot of the layer-wise CDIs, see Fig. S3C.

To further investigate the cell-class specific attentional modulation at each contrast level, we averaged AMI curves as a function of contrast across single units within a cluster (Fig. 3A *left*). The attentional modulation of $c_{50}$ and $n$ (Fig. 2F) suggests that medium- and broad-spiking neurons are more strongly modulated by attention in the range of low contrast than the range of high contrast. Indeed, we found that the AMIs of Medium and Broad clusters were relatively dependent on contrast, whereas the Narrow cluster is modulated in a contrast-independent manner (Fig. 3A *left*). When averaged across contrasts, attention positively modulated firing rates for all cell classes (Fig. 3A *right*, Wilcoxon signed rank test, *p* < 0.05 for all 3 clusters). Broad-spiking cells showed stronger attentional modulation than medium- and narrow-spiking cells (Wilcoxon rank sum test, Bonferroni corrected, n = 3, *p* < 0.0167). To quantify the contrast dependence of attentional modulation for each single unit, we first averaged the AMIs within the low-contrast and the high-contrast ranges with the threshold set at the best-fitting $c_{50}$ parameter. We then defined the contrast dependence index (CDI) of a single unit as the difference between the two average AMIs normalized by the AMI averaged across all contrasts (see *SI Text*). The sign of CDI value indicates the modulatory pattern of neuronal contrast response by attention: Contrast-independent modulation would result in CDI = 0, reflecting a pure scaling effect of attention on the CRF; A positive CDI implies a more robust attentional modulation in the low-contrast range. A negative CDI signifies a stronger attention effect in the high-contrast range (Fig. 3B). Therefore, for the further examination of CDI distributions, we focused on the statistical significance of their differences from 0 rather than their differences between each other. We reported any between-group comparisons in Table S2 of *SI Text*, but it is important to note that a statistical difference in CDI between groups cannot lead to an interpretation of distinct attentional modulation patterns. We inspected the CDI of neurons from each cell class and found that the mean CDI of the Narrow class was around zero. However, Medium and Broad clusters exhibited positive CDIs (Fig. 3C). These results are consistent with our findings of AMIs of CRF parameters for each cell class (Fig. 2F), confirming that attention modulated narrow-spiking neuron responses in a contrast-independent fashion, while attention effects on Medium and Broad classes depend on contrast and are more robust in the low-contrast range.

We further inspected the laminar profile of the attention effect and its contrast dependence for every cell class (Fig. 3D,E). When averaged across contrasts, narrow-spiking neurons showed significant attentional modulation only in the input layer, but not in the superficial or deep layer (Fig. 3D, right panels). On the other hand, the Broad cluster was robustly modulated by attention across all cortical layers (Fig. 3D, right panels). The AMI difference between these two cell classes is in agreement with the differences between narrow- and “broad”-spiking cells previously reported in these cortical layer^43^; it is important to note that the AMI pattern of the Medium cluster was different from that of the Broad cluster in the superficial layer, again implying that they represent distinct functional subpopulations (Fig. 3D, Wilcoxon rank sum test, Bonferroni corrected, n = 3, *p* < 0.0167). Two key laminar patterns of contrast dependence emerged from these three clusters. First, the attentional modulations of the Narrow and Medium clusters were independent of contrast across all cortical layers. Second, the Broad cluster exhibited strong contrast dependence and, specifically, significant modulation in the low-contrast range in the superficial and input layers; but its dependence on contrast was not significant in the deep layer (Fig. 3E). Notably, these laminar differences did not emerge when all units in a layer were analyzed as either a single class or more conventionally as narrow versus “broad” classes (Fig. S3B,C). Notwithstanding the limited number of units from individual subjects, we still observed the same CDI profiles of clusters in at least one monkey (Fig. S5I–L), supporting the robustness of the results.

### Laminar network mechanisms of contrast dependence of AMI across layers

We next used computational modeling to gain insights into the possible neural mechanisms underlying the layer- and cell-class specific AMI dependency on stimulus contrast. The normalization model was proposed as a unifying mechanism that could account for different forms of attentional modulation within a common computational framework^44^. It accounts for response gain and contrast gain effects by incorporating interactions between attentional gain and divisive normalization. The model is parsimonious in that it only contains three essential components: the stimulation field, the suppressive field, and the attention field (Fig. S4A). Variation in CDI across experimental paradigms^23,24,46^ has been explained by paradigm-specific stimulus size and attention field size in the normalization model^44^. However, the size of the stimulation field or the suppressive field in the model has not been examined in terms of their contributions to different forms of attentional modulation, which could be the potential neural mechanism underlying the layer-specific CDI in our empirical findings (Fig. 3E). To test this hypothesis, we varied the spread of the stimulation field or the suppressive field while keeping all other parameters unchanged (see *SI Text*). We then measured their effects on the attentional modulation of CRFs of output neurons. This inquiry was motivated by the observation that neuronal receptive field sizes change along the cortical depth in sensory areas^65–67^. Meanwhile, the stimulation field and the suppressive field in the normalization model are analogous to the receptive fields of excitatory and inhibitory neurons, respectively, when considering feedforward connections. The choice of other parameters was based on the fact that the stimulus size remained static throughout the recording sessions, and the assumption that the subject’s attentional strategy (i.e., the attention field) does not change for our experimental paradigm.

**Figure 4:**
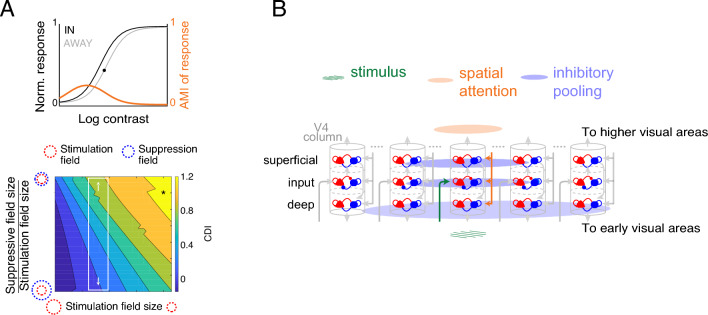
Computational Models Provide A Parsimonious Explanation for the Laminar Profile of AMI Contrast Dependence. (**A**) Predictions from the normalization model of attention with different suppressive field sizes or different stimulation field sizes. *Top*: Contrast response functions for a simulated neuron in the normalization model, when attending to a stimulus within the neuron's receptive field (black curve) and when attending toward the opposite hemifield (gray curve). The orange curve represents the AMI. The black dot shows the inflection point of “attend away” responses delimiting the low- and high-contrast ranges. *Bottom*: CDIs for simulated neurons as a function of the stimulation field size and the suppressive field size. The white rectangle depicts a potential mechanism that leads to the observed variation of CDIs across layers: changes in suppressive field size. The black asterisk corresponds to the model parameters used for the simulation in the top panel. (**B**) Proposed E–I networks in V4 accounting for the layer-specific CDI patterns. The empirical data and the model simulations imply a larger inhibitory pooling size in the deep layer compared to those in the superficial and the input layers. The arrows depict the canonical information flow pathways in a columnar circuit.

We simulated the normalization model with different sizes of the stimulation field or the suppressive field, generated neuronal contrast responses to an orientation with or without attention (Fig. 4A *top*), and computed the AMI as well as the CDI of a model neuron. We find that the CDI relies on the stimulation field size and the suppressive field size, both being inversely correlated with the CDI (Fig. 4A *bottom*). The pattern is robust regardless of the attention field size and the stimulus size (Fig. S4B,i). The results are also consistent across different input–output functions of the stimulation field (Fig. S4B,ii). Since broad-spiking neurons are putative excitatory pyramidal cells whose responses are modeled by the normalization model, these results suggest two possible neural mechanisms that explain the laminar profile of CDIs of broad-spiking neurons: the suppressive field size increases along the depth of V4 (Fig. 4A *bottom*) or the stimulation field is more extensive in the deeper layer of V4 (Fig. S4E *left*).

In the normalization model, connections leading to divisive normalization are feedforward, while the implementation of normalization can be feedforward or feedback or a combination of the two^44^. Under such assumptions, the stimulation field represents the receptive field of excitatory neurons, and the divisive suppression is mediated by local inhibitory interneurons. A direct test of the model predictions of the stimulation and suppressive fields sizes would be to examine the laminar receptive field sizes of excitatory neurons and inhibitory interneurons respectively. In the absence of such measurements for single units in our dataset, we chose to focus on other properties of neural spiking activities that are reflective of receptive field sizes, such as the spike-time correlation. The amplitude of the spike-time correlation between neurons has been shown to depend upon both the connection strength and the background synaptic noise^68^, both of which are related to the spatial range of inputs to the postsynaptic neuron. We hypothesized that a smaller receptive field of the postsynaptic neuron would make the local connections more dominant against background inputs and lead to a greater spike-time correlation between locally connected neurons.

To test this hypothesis, we set up a conductance-based spiking network model consisting of multiple local networks or “columns” of excitatory (E) and inhibitory (I) units that were mutually coupled (Fig. S4C *left*, S4E *middle*). In the interest of simplicity, one column of E and I neurons represents the local circuit in a cortical layer, and we modeled the spatial range of neuronal receptive fields in a column as the effective connections with excitatory neurons from other columns. We characterized the decay of between-column connections as a Gaussian function based on column distance whose standard deviation ($\sigma_{I}$ or $\sigma_{E}$) was used for controlling the receptive field sizes (Fig. S4C *left*, S4E *middle*). We simulated network spiking activity in response to a step input (Fig. S4C *middle*) and measured the spike-time correlation between local E and I populations within a column using pooled spike trains. The connection weights in the network were constrained by matching the amplitude of the network’s spike-time correlations with the empirical findings. We find that the inhibitory receptive field size has a critical impact on the spike-time correlation amplitude in such a network (Fig. S4C *right*), while the excitatory receptive field size has little effect (Fig. S4E *right*). A larger inhibitory receptive field (larger values of $\sigma_{I}$) leads to a lower spike-time correlation between the local E and I populations in the network. This result suggests that changes in the inhibitory receptive field size down the cortical depth could be revealed by comparing the magnitudes of spike-time correlation between E and I populations among layers.

Driven by the conclusion from the spiking network model, we computed the session-averaged spike-time correlation between Narrow (putative inhibitory neurons) and Broad (putative excitatory neurons) classes within each layer (see *SI Text*) in our empirical data. The correlation amplitude was higher in the superficial layer and the input layer than that in the deep layer (Fig. S4D). This was confirmed by comparing their mean spike-time correlations within a 50-ms time window (Fig. S4G). These findings from the model E–I network (Fig. S4C) support our normalization model-based prediction that inhibitory neurons in the deep layer exhibit relatively broader receptive fields, which in turn generates lower CDI values of broad-spiking neurons (Fig. 4A *bottom*). Our findings thus provide a parsimonious explanation for the layer- and cell-class specific contrast dependence of attentional modulation observed in area V4 (Fig. 4B).

## Discussion

Spatial attention plays a critical role in sensory guided behavior. It is thought to achieve this by boosting overall responses or by selectively enhancing the responses to low contrast stimuli in mid-tier visual cortical areas such as V4. While later stages of the visual processing hierarchy are thought to benefit from the contrast gain effect, V4 also sends feedback projections to early visual areas that use veridical representation of contrast to aid object recognition, and are modulated by attention in a contrast-independent manner^26,27^. How area V4 meets these distinct information processing demands has remained unresolved. Contrary to the simplifying assumptions of prior empirical studies, we tested the hypothesis that V4 customizes its output to different stages of the visual processing hierarchy through layer- and cell-class specific attentional modulation of contrast computations. Recent advances in experimental techniques have shown layer- and cell-class specific functional specificity of computations in the cortical circuit^33,69–71^. However, these studies have been limited to species in which higher cognitive functions, such as attention, are challenging to study (but see^72^). Using computational approaches on laminar neural data in area V4 of the macaque, we find that the attentional modulation of neural responses to visual luminance contrast is indeed layer- and cell-class specific. We classified neurons into three functional cell classes defined by their action potential widths (Fig. 2B); these classes show specificity in their response time to visual input (Fig. 2E), attention effects on their contrast response functions (Fig. 2F) and the contrast dependence of attentional modulation (Fig. 3C). Specifically, narrow- and medium-spiking neurons show contrast-independent response modulation across layers; broad-spiking neurons, the putative projection neurons, exhibit significant contrast dependence of attentional modulation in the superficial layers, that project to higher level visual areas, but not in the deep layers, that project to earlier visual areas (Fig. 3D,E). Notably, this significant laminar difference was not observable without cell-class identification (Fig. S3B,C). These results provide the first evidence for our broad hypothesis that attentional modulation of contrast computations in the visual cortex is heterogeneous across those cell classes and layers that project to distinct stages of the visual processing hierarchy. The qualitative nature of the attention modulation of contrast in our data is not only distinct but suggests optimization for the computational demands of the target stages. Attentional boosting of responses in projection populations of superficial and deep layers shows two modes. Superficial layers that principally project representations such as extended contours and object surfaces to higher areas (see^25^ for a review) are characterized by selective boosting of responses to low contrast stimuli. On the other hand, contrast-independent scaling of neural responses is confined to the deep output layers. Neurons in these layers project back to early visual areas that are reliant on faithful representation of luminance contrast for low-level feature extraction. We speculate that the contrast-independent attentive feedback provides a spatial boost signal to early visual areas that do not receive direct inputs from attention control centers such as the frontal eye fields^30^. This also aligns with the predictive coding model of object recognition, wherein V4 is a higher-level area in the object recognition hierarchy that generates predictions of lower-level activity, without corrupting the sensory landscape that is needed for error correction^73^.

When interpreted within the framework of the normalization model of attention (Fig. 4A), the layer-specific attention modulation predicts differences in the spatial pooling of local inhibitory populations across layers. Such differences further predict a layer-specific signature of correlations between the activities of local inhibitory and putative excitatory neurons when explored in a spiking E–I network model (Fig. S4C). We find robust evidence for differences in inhibitory spatial pooling across layers through our analyses of correlations between putative inhibitory and putative excitatory neurons in the superficial, input, and deep layers of the cortex (Fig. 4B, S4D).

### Origin of contrast-dependent attentional modulation

Whether V4 is in a unique position to strengthen the perceptual contrast in the attentive state remains a question for future empirical studies. The positive mean CDI value of broad-spiking cells in the superficial layer meets the functional expectation that the contrast-invariant representations in the higher visual areas benefit from the feedforward projections principally originating from the V4 superficial layer. We also observed a positive mean CDI value in the input layer of V4, which suggests that the contrast gain effect could either be implemented by the local circuit in the V4 input layer or be inherited from earlier visual areas. Given the contrast-invariant attention effect observed in V1^26,27^, we have a higher confidence to believe that the contrast gain computation is realized in the V4 input layer. However, this hypothesis needs to be tested by doing a similar analysis of attention modulation in other early visual areas such as V2.

Anatomical evidence suggests that in V4, neurons in the superficial layer can also produce feedback connections and neurons in the deep layer can contain the feedforward pathway^74^. Therefore, one might argue that inter-area connections of the superficial and deep layers might not be so different that distinct attentional modulations are required. However, according to^74^, both superficial and deep layers exhibited high topological precision: most neurons had point-to-point connectivity and only a small fraction of neurons were dual-labeled. Thus, most projections from superficial and deep layers of V4 target separate individual cells in other visual areas. Although further work is required to answer the question, it is likely that these differentially targeted neurons carry out distinct functions in the target regions based on their attentional modulations shown by our study. Therefore, our conclusion still stands in terms of the functional compartmentalization of attention effects on contrast response function. For example^27^, found that attentional modulation in V1 was relatively independent of the stimulus contrast, suggesting that the neuronal population they recorded were likely targeted by feedback projections from the deep layer of V4.

### Classification of cell-types

The duration of the extracellular spike waveform has been used to distinguish putative inhibitory interneurons from putative excitatory pyramidal cells in a wide range of species and across various brain regions^14,43,49,54,56,75–81^. In a study using single tungsten electrodes, narrow-spiking neurons showed stronger attention-dependent increases in absolute firing rates than broad-spiking cells, but the percentage of increase did not significantly differ across the two populations^14^. We found that narrow-spiking neurons had comparable attentional modulation as broad-spiking neurons only in the input layer (Fig. 3D). We speculate that data collected from single tungsten electrodes might bias the data towards units with higher firing rates that are more likely to reside in the input layer.

Unsupervised clustering algorithms are also effective in identifying subpopulations of neurons with distinct functional properties^56,82–84^. One non-human-primate study classified neurons purely based on tuning properties^83^. Others also took the waveform into account^56,84^. Our classification is different from these previous attempts in two ways. First, the peak-to-trough duration is the only feature in our cluster analysis, which renders our clusters easier to interpret. Second, we do not assume the bimodal distribution of the PTD distribution like previous studies^56,84^. The emergence of medium-spiking neurons was data-driven and robustly validated by criteria for model selection.

It is important to note that the clusters we distinguished based on spike width may not simply correspond to neuronal classes differentiated based on morphology or protein expression patterns^85–87^. Potential mapping exists between the narrow-spiking neurons and inhibitory interneurons, and between the broad-spiking neurons and pyramidal cells^58–60^. However, growing evidence suggests a more complicated relationship. For example, in the range of inhibitory interneurons, parvalbumin-expressing (PV+) neurons are shown to possess a narrow spiking phenotype, somatostatin (SOM) expressing (SOM+) neurons, which correspond to calbindin-expressing (CB+) neurons in primate, show both narrow and broad spiking phenotypes, vasoactive intestinal peptide expressing (VIP+) neurons, which correspond to calretinin-expressing (CR+) neurons in primate, show predominantly broad spiking property^88^. Evidence of a “thin spiking” glutamatergic class in the macaque motor cortex suggests a mixed excitatory-inhibitory composition of Narrow class in the motor cortex^89^. Moreover, these different interneuron types display non-uniform distributions across laminar locations^88,90^. Therefore, to inform a comparable effect on the composition of Narrow population in a layer and area-specific manner in the future, a combined molecular and electrophysiological characterization in the visual cortical areas in a species-specific manner is needed^91^. On the other hand, there is a robust agreement across species on the excitatory nature of the units we categorized as Broad, and which inform the main thesis of our study. We find differences in response latencies (Fig. 2E, S2B), firing rate (Fig. S2A) and attentional modulation of firing rates (Fig. 3A,D) between clusters, suggesting their different functional roles in attention-mediated visual processing. Crucially, these distinct functional roles are reflected by the differences in contrast dependence of attentional modulation.

### Circuit roles for identified cell classes

The response time analysis (Fig. 2E, S2B) in our study revealed potential roles for the three cell classes in the local circuit. Medium-spiking neurons, with their shortest response times in all three layers, suggest neurons receiving feedforward inputs in every laminar compartment. The intermediate spike width of this cluster is comparable to both excitatory and at least two inhibitory cell types—SOM + and VIP+^92,93^—in the rodent, and CB+ and CR+ cells^88^ in primate. However, little evidence exists for these inhibitory subtypes to be recipients of direct feedforward inputs as suggested by the fast response time in our data. Broad-spiking neurons, which exhibit the longest average neuronal latencies across layers, might play a role in integrating lateral and feedback inputs and act as projection neurons in both the superficial and deep layers due to their maximal overall attentional modulation. Narrow-spiking cells, given their intermediate response time, might represent inhibitory interneurons that are mainly driven by recurrent excitatory inputs, and provide inhibition to local neurons. Here, our response time findings differ from those in prior studies in primary sensory areas, where the fastest response time was identified for fast-spiking inhibitory neurons with narrow spike width and/or PV+ labeling^92–94^, and which are driven by feedforward inputs. Two possible scenarios could explain these differences. First, the balance of feedforward vs. feedback processing in local inhibitory neurons could be different across areas. Such differences could be reflected in the temporal emergence of feature tuning in neural responses: earlier in primary areas such as V1 and later in tertiary areas such as V4^95^. Second, the excitatory/inhibitory subtype makeup of narrow spiking units could differ between areas; for example, in addition to fast-spiking inhibitory neurons, a subtype of layer-5 pyramidal neurons in motor cortex also exhibits narrow spike width^89^ as noted above. Whether such populations exist in area V4 remains to be investigated.

### Relation to prior studies of spatial attention in V4

Prior studies evaluating attention effects on neuronal contrast responses proposed either contrast-independent scaling of responses, termed as response gain^11,96–98^ or boosting of responses to low contrast stimuli, termed as contrast gain^23,24,99,100^ or an intermediate effect between the two^46,101^. However, the studies that concluded that their data support the claim that attention changes response gain, nevertheless saw larger percentage increases with attention, on average, at low contrast, than at high contrast^46^. Computational modeling showed that both contrast- and response-gain patterns were consistent with the normalization model of attention, and that the two distinct conclusions could be explained by differences in the task and stimuli used in the relevant studies^44^. Our characterization of attention effects in terms of the CDI offers an exciting and more nuanced alternative explanation for the differences reported by these previous studies, in a neural subpopulation specific manner. Although the overall attentional modulation in undifferentiated population in our dataset is consistent with the intermediate effect (Fig. 1F, S1C), attention effects on individual clusters are distinct: a mixture effect of response gain and contrast gain is observed for medium- and broad-spiking units (with differences in $c_{50}$ modulation); narrow-spiking cells show a response gain change (Fig. 3A). Furthermore, broad-spiking neurons exhibit larger attention-dependent increases in response than the population mean, especially within the low-contrast range (Fig. 3A). This observation suggests that attentional modulation of firing rate for broad-spiking cells may be more robust than that gleaned from previous studies which averaged across the whole recorded population. These cell-class specific increases in firing rate may significantly improve the signal-to-noise ratios of individual neurons, and therefore, act as another important contributor to the improvement of psychophysical performance due to attention in addition to reductions in correlations^13,15^. Finally, our results take us an important step forward, by showing how these different patterns vary by layer. For example, broad-spiking neurons in the deep layer show a CDI pattern reflective of response gain, whereas a mixture of response gain and contrast gain appears for the same cluster type in other layers (Fig. 3D,E). These layer-specific differences could contribute targeted computations to the different levels of visual processing hierarchy served by each layer.

### Our interpretation of the normalization model

The predictions from the normalization model (NM) of attention provide one possible explanation for the diverse contrast modulation patterns across layers. The NM assumes that both stimulus parameters and attention condition contribute to the normalization input to local excitatory neurons and lead to different patterns of attentional modulation across studies. The stimuli presented in our experiments were optimized for the recording site and did not change with attention condition, and hence are not assumed to contribute differentially to the normalization mechanism. The attention field in NM describes the attention gain for each neuron in the population and depends on the animal’s attentional strategy employed during the experiment^102^. The neural substrate for the attention field is unspecified in the NM, but we assumed the attention field to be constant across the cortical depth since the data was collected using a fixed experimental paradigm. However, given our current lack of understanding of the biophysical mechanisms underlying attentional modulation, our interpretation of the attention field may be subject to future revision. The extent of excitatory receptive field, also termed as the stimulation field, in the NM can be mediated by various cortical connectivity patterns. While we explored a lateral pooling mechanism as the determinant in the spiking network model, innervation specificity of feedforward synaptic input could be an alternative mechanism^75,103^.

The variation in contrast dependence of attentional modulation observed across layers and cell classes (Fig. 3D,E) in our data is explained by the NM in a most parsimonious way via the variability of the suppressive field size (Fig. 4A). However, the NM is agnostic to the neural machinery dedicated to the formation of neuronal tunings or the implementation of attentional modulation. To explore the implications of its field size predictions on spike-time correlations in a biophysical model, we considered the model’s stimulation field as the receptive field of putative excitatory projection neurons in a column, and its suppressive field as the receptive field of local inhibitory interneurons. It is important to note that our model is not a spiking network implementation of the entirety of attention computations described by the NM. The suppressive field in NM, which mediates divisive normalization, is a computation that can be can be implemented through a variety of mechanisms^44^. We have explored one of the candidate suppression mechanisms—pooling of lateral inputs by local inhibitory interneurons^104–106^. A feedforward mechanism of variable suppressive fields would yield a very similar prediction for spike-time correlations between local E and I populations. When testing the model’s predictions in our dataset, we ascribed the suppressive field to the receptive field of the Narrow cluster (putative interneurons), and we regard the stimulus drive as the Broad cluster, but the Medium cluster also showed a similar laminar pattern of spike-time correlations (Fig. S4D,F).

Note that our spiking neural network model was based on a fixed ratio of excitatory and inhibitory neurons (4:1). However, the proportion of interneurons varies across sensory and executive areas. For example, PV+ neurons that operate directly on excitatory cells are the most abundant interneuron type in MT while CR + which mainly mediates disinhibition is the most abundant one in the prefrontal cortex^88^. Therefore, to reveal the effects of inhibitory pooling size on neuronal CCGs in other cortical areas, the simulation of spiking neural networks should adapt to the specific interneuron proportions in the region of interest.

## Materials and methods

### Electrophysiological recording

V4 neuronal responses were collected in two adult male rhesus macaques (Macaca mulatta) during an attention-demanding orientation detection task using 16-channel linear array electrodes (Plexon, Plexon V-probe) or single tungsten microelectrodes (FHC Inc). All procedures were approved by the Animal Care and Use Committee at the Salk Institute for Biological Studies and conformed to NIH guidelines. Neural firing rates in response to a particular contrast were measured by counting the number of spikes within a period of 60–260 ms after stimulus presentation. See *SI Text* for additional details.

### Analysis and modeling

We classified single units using unsupervised learning algorithms based on the spike shape (see *SI Text*). We explored the neural mechanisms of attentional modulation within the framework of the normalization model of attention^44^. We manipulated the stimulation and suppressive field sizes and computed the attention modulation of model neurons for every condition. For studying the effect of receptive field size on spike-time correlations, we utilized a spiking network model of cortical columns (see *SI Text*).

### Ethics

The animal experiments were performed in strict accordance with the recommendations in the Guide for the Care and Use of Laboratory Animals of the National Institutes of Health. All animals were handled according to approved institutional animal care and use committee (IACUC) protocols of the Salk Institute. All procedures were approved by the Institutional Animal Care and Use Committee at the Salk Institute (Protocol #14-00014) and conformed to NIH guidelines. All studies were reported in compliance with the ARRIVE guidelines (Animal Research: Reporting In Vivo Experiments).

### Supplementary Information

Supplementary Information 1.

Supplementary Figures.

## Data Availability

The datasets used and analyzed during the current study are available from the corresponding author on reasonable request.
