# Supplementary material for: Laminar compartmentalization of attention modulation in area V4 aligns with the demands of visual processing hierarchy in the cortex
Source: Sci Rep. 2023 Nov 9;13:19558. doi: 10.1038/s41598-023-46722-8 (PMC10636153; doi:10.1038/s41598-023-46722-8)
Supplement: Supplementary file 1 — Supplementary Information 1. [file 41598_2023_46722_MOESM1_ESM.docx]

**SI Text**

**Surgical Procedures.** The surgical procedures, recording configurations, and task design have been described in detail in previous studies^43,105^. In brief, an MRI compatible low-profile titanium chamber was placed over the pre-lunate gyrus, on the basis of preoperative MRI imaging in two rhesus macaques (right hemi-sphere in Monkey A, left hemisphere in Monkey C). The native dura mater was then removed, and a silicone based optically clear artificial dura (AD) was inserted, resulting in an optical window over dorsal V4. All procedures were approved by the Animal Care and Use Committee of the Salk Institute for Biological Studies and conformed to NIH guidelines.

**Electrophysiological Recording.** At the beginning of each recording session, we inserted 16-channel linear array electrodes (“laminar probes”, Plexon, Plexon V-probe) or tungsten microelectrodes (FHC Inc) through the artificial dura into cortical sites near the center of the prelunate gyrus. Neuronal signals were recorded, filtered, and stored using the Multichannel Acquisition Processor system (Plexon). Neuronal signals were classified as either isolated single units or multiunit clusters by the Plexon Offline Sorter program. For the data collected from linear array electrodes, we used current source density analysis^106^ to identify the superficial (Layers 1-3), input (Layer 4), and deep (Layers 5 and 6) layers of the cortex based on the second derivative of the flash-triggered LFP^43,105^. Cell bodies of single units with bi-phasic action potential waveforms were assigned to the same layer in which the electrode channel was situated during recordings. We only considered units as potential candidates when their waveforms had a clearly defined peak *preceded* by a trough. Units that had tri-phasic waveforms or other shapes were excluded from analyses. Valid single-unit extracellular data were collected over 29 sessions (20 sessions in monkey A, 9 in monkey C) using laminar electrodes and 37 sessions (21 sessions in monkey A, 16 in monkey C) using tungsten electrodes, yielding 410 single units in total (337 units from linear array electrodes and 73 units from tungsten electrodes). Unit yield per session was considerably higher in monkey C than monkey A, resulting in a roughly equal contribution of both monkeys toward the population data.

**Attention Task and Stimuli.** Well-isolated single units were recorded from area V4 of two rhesus macaques during an attention-demanding orientation change detection task (Figure 1A).

*Receptive field mapping.* At the beginning of each recording session, we mapped neuronal RF’s using subspace reverse correlation with Gabor (eight orientations, 80% luminance contrast, spatial frequency 1.2 cycles/degree, Gaussian half-width 2°) or ring (80% luminance contrast) stimuli presented at 60 Hz while monkeys maintained fixation. Each stimulus appeared at a random location selected from an 11 × 11 grid with 1° spacing in the appropriate visual quadrant. Spatial receptive maps were obtained by applying reverse correlation to the time-averaged power in the evoked LFP signal (0-200 ms after each stimulus flash) at each recording site. The resulting spatial map of LFP power was taken as the spatial RF at the recording site. All RFs were in the lower visual quadrant (lower left in monkey A and lower right in monkey C) and with eccentricities between 2 and 7 dvas. The tuning properties of the neuron (for single tungsten recordings) or of the aggregate column (for laminar recordings) were estimated during RF mapping. The orientation selected for the main attention task corresponded to the estimated peak of the tuning curve.

*Attention task.* While the monkey maintained fixation, two oriented Gabor stimuli (orientation optimized for each session, spatial frequency 1.2 cycles/degree, size scaled to three quarters of the estimated diameter of the V4 neuron receptive field) were flashed on for 200ms and off for variable intervals (randomly chosen between 200 and 400ms). The contrast of each flash of stimulus was randomly chosen from a uniform distribution of 6 contrasts (c = [10%, 18%, 26%, 34%, 42%, and 50%]). One of the stimuli was located at the receptive field overlap region of the recorded neurons and the other at an equally eccentric location across the vertical meridian. At the beginning of a block of trials, the monkey was spatially cued to covertly attend to one of the two spatial locations using instruction trials in which only one stimulus was presented. One of the two stimuli changed in orientation at an unpredictable time (minimum 1s, maximum 5s, mean 3s). The monkey was rewarded for making a saccade to the location of orientation change. 95% of the orientation changes occur at the cued location, and 5% occur at the uncued location (foil trials). We observed impaired performance and slower reaction times for the foil trials, suggesting that the monkey was indeed using the spatial cue to perform the task. The difficulty of the task was controlled by changing the degree of orientation change (randomly chosen from the following: 1°, 2°, 3°, 4°, 6°, 8°, 10°, and 12°). If no change occurred before 5s, the monkey was rewarded for holding fixation (catch trial, 13% of trials).

**Contrast Response Function (CRF).** Neuronal responses were analyzed only for correctly performed trials, excluding instruction trials. We restricted all data analysis to non-target stimuli because neuronal responses to target stimuli were generally affected by the behavioral response or the reward delivery, which occurs on correct trials after the target's appearance. Moreover, the larger number of non-target stimuli compared to target stimuli provided a more reliable response strength measure. For both attention conditions, the firing rate of a single unit in response to a particular contrast was measured by counting the number of spikes within a period of 60-260ms after stimulus onset. Its baseline firing rate in each attention condition was extracted from a 200ms window before a stimulus flash. The mean firing rates and the standard deviations (SDs) were generated across all stimulus flashes.

We adopted the bootstrap procedure to prevent overfitting to the available activity data^107^, we drew 1000 random samples of contrast responses from a normal distribution with the same mean and standard deviation as the experimental data for each visually responsive single unit. For each attention condition, we computed the CRF for each random sample by applying an ordinary least square fit to a hyperbolic ratio function:

$$\begin{aligned} R= R_{max} \cdot\frac{c^{n}}{c^{n}+ c_{50}^{n}}+m\#(1) \end{aligned}$$

where $R$ is the neuronal response, $R_{max}$ is the maximum attainable response, $c$ is the contrast, $c_{50}$ is the contrast at which response is half-maximal, $m$ is the baseline activity, and $n$ describes the steepness of the response function and represents the neuron’s sensitivity to contrast. This function has been shown to provide a good ﬁt to contrast response functions from visual cortices in cat and macaque monkey^46,108^. We then averaged the best-fitting CRFs across random samples to generate the mean CRF for each visually responsive single unit (Figure 1C).

**Inclusion Criteria.** We used all 410 single units (337 units from laminar recordings and 73 units from single-electrode recordings) for clustering (Figure 2B), but we only included neurons that were visually responsive for further analysis. We considered a neuron as visually responsive if any contrast responses exceeded its baseline firing rate by 4 SDs for both attention conditions. 255 of 410 single units (198 units from laminar recordings and 57 units from single-electrode recordings) were significantly driven by the task stimuli. We computed the firing rates and attentional modulation of CRF on all 255 single units (Figure 1E, 1F, 2F, 3A, S1C, S2A, S2D, S3A). For contrast dependency analysis (see **Attentional Modulation Index and its Contrast Dependency**, Figure 3C), we removed the outliers of the index distribution within each cluster to aid the visualization. We considered a value as an outlier if it was more than 1.5 times the interquartile range away from the 25th and 75th percentiles of the distribution. This resulted in 217 single units in total (172 units from laminar recordings and 45 single-electrode recordings). For response latency analysis (see **Firing Rate Estimation and Response Time**, Figure 2E, S2B, S2C, S5E, S5F), we only included a subset of 255 neurons that had at least 20 spikes within the 200ms pre-stimulus time windows to reliably estimate firing rate curves. Within each cluster, we first removed outliers as described above, and then removed those that did not meet the response time criteria as detailed below, which led to 175 single units (143 units from laminar recordings and 32 units from single-electrode recordings) for the variable kernel bandwidth and 214 single units (167 units from laminar recordings and 47 units from single-electrode recordings) for the fixed kernel bandwidth. We excluded all units from single-electrode recordings for laminar analyses (Figure 3D, 3E, S2A, S2B, S3B, S3C, S5K, S5L).

**Firing Rate Estimation and Response Time.** To measure a neuron’s response time to stimulus presentation, we combined spike trains across stimulus flashes for each single unit beginning 200ms prior to stimulus onset and ending 260ms after stimulus onset. We then convolved each spike with a Gaussian kernel and computed the average firing rates across flashes for each contrast level. The kernel bandwidth was variable (Figure 2E, S2B) at each time point or was fixed (Figure S2C) unit-by-unit (selected from 1-10ms). The value was selected by a bandwidth optimization algorithm that minimized the mean integrated squared error between the kernel estimate and the unknown underlying density function^64^. Since attention barely affects neuronal latencies^109^, we included trials from both “attend-in” and “attend-away” conditions to maximize the number of spikes. We only included units that fired at least 20 spikes within 200ms before stimulus onset to ensure reliable firing rate estimates. We also visually inspected the firing rate estimates of all neurons and removed those with noisy responses. For the variable kernel bandwidth, the response time of a unit to the stimulus was defined as the delay after stimulus onset at which its average firing rate exceeded 2 times of the 95% bootstrap confidence interval of the pre-stimulus period. For the fixed kernel bandwidth, we defined the response time as the first time bin after stimulus onset that reached a firing rate higher than the maximum of the pre-stimulus period and persisted for at least five time bins^50^.

**Clustering Analysis.** We used the *k*-means clustering algorithm^57^ and a meta-clustering analysis^56^ to characterize cell classes based on peak-to-trough duration (PTD): we ran 500 realizations of the *k*-means for each *k* and selected the best replicate from 50 replicates for each realization. After 500 realizations of each *k*, we computed the probability that pairs of neurons belonged to a same cluster. Valid clusters were identified by setting a probability threshold (*p* ≥ 0.9). We considered clusters with at least five single units as reliable.

We computed the Akaike information criterion (AIC) and the Bayesian information criterion (BIC) to estimate the quality of clustering:

$$\begin{aligned} AIC= -2\sum_{i=1}^{k} L_{i}+4km \#\left( 2 \right) \end{aligned}$$

$$\begin{aligned} BIC= -2\sum_{i=1}^{k} L_{i}+2km\cdot\ln\left( n \right)\#\left( 3 \right) \end{aligned}$$

where $n$ is the total number of units, $m$ is the dimension of data in a being considered $k$-cluster solution ($m=1$ in our study) and $L_{i}$ is the log-likelihood of sum of squares of cluster $i$.

$$\begin{aligned} L_{i}= -n_{i}\ln\left( \sum_{r=1}^{m} \sum_{j=1}^{n_{i}} \left( y_{r}^{j}- \bar{y_{r}} \right) \right)\#\left( 4 \right) \end{aligned}$$

where $n_{i}$ is the number of units in cluster $i$, $y_{r}^{j}$ is the $r^{th}$ feature value of unit $j$, $\bar{y_{r}}$ is the mean of the $r^{th}$ feature in cluster $i$.

To identify the most appropriate number of clusters, we implemented the Kneedle algorithm^110^ to detect the point of maximum curvature (elbow point) in both AIC and BIC curves. The algorithm normalizes the points of the curve to the unit square and computes the set of differences between the $x$- and $y$-values, *i.e.*, the set of points ($x$, $y-x$) as in Figure 2A. An elbow point is then defined as the local maximum of the difference curve. Both AIC and BIC curves suggest that $k=3$ is the optimal number of clusters for our dataset (Figure 2A).

**Attentional Modulation Index and its Contrast Dependency.** The attentional modulation index (AMI) of a neuron during the stimulus presentation with a specific contrast $c$ was calculated using the best-fitting contrast response functions ($r$) from both attention conditions:

$$\begin{aligned} AMI\left( c \right)=\frac{{r\left( c \right)}^{IN}-{r\left( c \right)}^{AWAY}}{{r\left( c \right)}^{IN}+{r\left( c \right)}^{AWAY}}\#\left( 5 \right) \end{aligned}$$

The contrast dependence of the AMI was measured by the contrast dependence index (CDI):

$$\begin{aligned} CDI=\frac{\bar{{AMI}_{low}}-\bar{{AMI}_{high}}}{\left| \bar{{AMI}_{all}} \right|}\#\left( 6 \right) \end{aligned}$$

where $\bar{{AMI}_{low}}$ and $\bar{{AMI}_{high}}$ are the average AMIs within the low-contrast range and the high-contrast range, respectively. $\bar{{AMI}_{all}}$ is the average AMI across all contrasts. $c_{50}$ from the best-fitting CRF during “attend away” condition delimited the range of low contrast (${c<c}_{50}$) and the range of high contrast (${c\geq c}_{50}$). CDI measures how the AMI of a neuron fluctuates with the contrast of the stimulus. A zero CDI indicates that the AMI is independent of the contrast of the stimulus. More robust attentional modulation at the low-contrast range leads to positive CDIs, and more potent attention effects at the high-contrast range result in negative CDIs (Figure 3B).

**Normalization Model Simulations.** We used the normalization model of attention^44^ to explore the neural mechanisms behind the variety of attentional modulation across layers (Figure S4A). The normalization model posits that the resulting firing rate ($R$) of simulated neurons can be produced from a product of the stimulus drive ($E$) and the attention field ($A$), normalized by the suppressive drive ($S$):

$$\begin{aligned} R\left( c;x,\theta\right)=\frac{A\left( x,\theta\right)E\left( x,\theta;c \right)}{S\left( x,\theta;c \right)+\sigma}\#\left( 7 \right) \end{aligned}$$

where $x$ and $\theta$ represent the receptive field center and orientation preference of each neuron in the population. $c$ is stimulus contrast and $\sigma$ is a constant that controls the contrast gain of the neurons’ response. The stimulus drive is the output of the stimulation field, which represents the range of space and orientations that evokes excitatory responses. The attention field describes the strength of attentional gain in the spatial and feature spaces, whose value is 1 for unattended space and is greater than 1 for the attended stimulus. The suppressive drive is the output of the suppressive field, which represents the range of space and orientations that suppress the responses and receives feedforward stimulation drive modulated by attention:

$$\begin{aligned} S\left( x,\theta;c \right)=s\left( x,\theta\right)*\left[ A\left( x,\theta\right)E\left( x,\theta;c \right) \right]\#\left( 8 \right) \end{aligned}$$

where $s\left( x,\theta\right)$ is the suppressive field and $*$ represents convolution. The stimulus, stimulation field, attention field, and suppressive field all had Gaussian profile in space and orientation.

Important hyperparameters of the normalization model include the stimulus size, the attention field size, the stimulation field size, the suppressive field size, and the orientation tunings of the three fields. The stimulus size and the attention field size have been shown to be critical factors in shaping the forms of attentional modulation^44^. But the effects of the stimulation field size or the suppressive field size have not been explored. We listed all hyperparameters we changed in Table S1.

Table S1. Parameters of the normalization model of attention

| Figure Panel | Stimulation Field Size (relative to the attention field) | Suppressive Field Size (relative to the stimulation field) | Attention Field Size | Stimulus Size |
| --- | --- | --- | --- | --- |
| 4A | 1/3 - 2 | 1 - 6 | 30 | 5 |
| S4B, i | 1/6 - 2 | 1 - 10 | 10, 20, 30 | 5, 10, 20 |
| S4B, ii | 1/6 - 2 | 1 - 10 | 30 | 5 |

The stimulation field size was varied based on its ratio relative to the attention field size, and the suppressive field size was changed based on its ratio relative to the stimulation field size. The orientation tuning widths of the stimulation field and the attention field were both 60°, and the tuning width of the suppressive field was 360°. A baseline activity of 0.5 was added after the normalization. For each combination of parameters, the AMI at each contrast level was calculated using the model neuron responses from two attention conditions. The CDI was computed from the AMIs averaged within the low-contrast range or the high-contrast range delimited by the CRF’s inflection point from the “attend away” condition.

For simulations in Figure S4B, we further modified the input-output (I-O) function of the stimulus drive from a linear function to a nonlinear sigmodal function:

$$\begin{aligned} r\left( c \right)=\frac{c}{c+\sigma} \#\left( 9 \right) \end{aligned}$$

where $\sigma$ is 0.26, matching the average $c_{50}$ of our data. We also applied either a multiplicative response gain (10% of increase in overall response) or a contrast gain (1% of increase in perceived contrast $c$) to the I-O function to confirm that the CDI profile generated from the model is independent from the types of the stimulus drive input.

**Spiking Network Model.** *Model Structure:* We set up a conductance-based model of $N_{E}=800$ excitatory (E) and $N_{I}=200$ inhibitory (I) neurons with a connection probability of 0.5. Neurons were evenly divided into 10 columns or local sub-networks around a ring with the following within-column synaptic weights:

E to E : $w_{EE}=\frac{W_{EE}}{N_{E}}$; I to I : $w_{II}=\frac{W_{II}}{N_{I}}$; E to I : $w_{IE}=\frac{W_{IE}}{N_{E}}$; I to E : $w_{EI}=\frac{W_{EI}}{N_{I}}$

The weights were chosen to match the empirical cross-correlations: $W_{EE}=10, W_{II}=1, W_{IE}=6, W_{EI}=18$.

To describe the receptive field size in simplicity, we only modeled E to I connections and E to E connections between different columns. The synaptic weights fell off with column distance following a Gaussian profile:

$$\begin{aligned} w^{ij}=\frac{W}{N_{E}}\times\frac{1}{\sigma\sqrt{2\pi}}\exp\left( -\frac{1}{2}\left( \frac{d_{ij}}{\sigma} \right)^{2} \right)\#\left( 10 \right) \end{aligned}$$

where $w^{ij}$ is the synaptic weight between two columns ($w_{IE}^{ij}$ or $w_{EE}^{ij}$) and $d_{ij}$ represents the distance from column $j$ to column $i$. $\boldsymbol{\sigma}$ controls the receptive field size of the postsynaptic inhibitory ($\sigma_{I}$) or excitatory ($\sigma_{E}$) neuron.

*Neuron Model:* The neuron model aimed for producing regular spiking dynamics. Spiking units were modeled as Izhikevich neurons^111^ which exhibit firing patterns with high computational efficiency:

$$\begin{aligned} \frac{dv}{dt}=0.04v^{2}+5v+140-u+I\#\left( 11 \right) \end{aligned}$$

$$\begin{aligned} \frac{du}{dt}=a\left( bv-a \right)\#\left( 12 \right) \end{aligned}$$

$$\begin{aligned} if v \geq30 mV, then \left\{ \begin{aligned} v \leftarrow c \\ u \leftarrow u+d \end{aligned} \right.\#\left( 13 \right) \end{aligned}$$

$v$ represents the membrane potential of the neuron and $u$ is a membrane recovery variable. $I$ is the current input to the neuron (synaptic and injected DC currents). We picked hyperparameters $a$, $b$, $c$, and $d$ to produce simple firing patterns:

Regular spiking excitatory units: $a=0.02, b=0.2, c=-65, d=8$

Fast spiking inhibitory units: $a=0.1, b=0.2, c=-65, d=2$

Presynaptic excitatory neurons generate fast (AMPA) and slow (NMDA) synaptic currents, while presynaptic inhibitory neurons generate fast GABA currents:

$$\begin{aligned} I_{syn}= \sum_{i} g_{AMPA}\left( t \right)\left( v\left( t \right)- V_{AMPA} \right)+ \sum_{j} g_{NMDA}\left( t \right)\left( v\left( t \right)-V_{NMDA} \right) \\ + \sum_{k} g_{GABA}\left( t \right)\left( v\left( t \right)- V_{GABA} \right)\#\left( 14 \right) \end{aligned}$$

where $V_{AMPA}=0$, $V_{NMDA}=0$, $V_{GABA}=-70$ are the respective reversal potentials (mV). The synaptic time course g(t) was modeled as a difference between exponentials:

$$\begin{aligned} g\left( t \right)= \frac{1}{\tau_{d}-\tau_{r}}\left[ exp\left( -\frac{t-\tau_{l}}{\tau_{d}} \right)-exp\left( -\frac{t-\tau_{l}}{\tau_{r}} \right) \right]\#\left( 15 \right) \end{aligned}$$

where the parameters $\tau_{d}, \tau_{r},$and $\tau_{l}$ are the decay, rise, and latency time constants with the following values:^112^ AMPA: $\tau_{d}=2$ ms, $\tau_{r}=0.5$ ms, $\tau_{l}=1$ ms; NMDA: $\tau_{d}=80$ ms, $\tau_{r}=2$ ms, $\tau_{l}=1$ ms; GABA: $\tau_{d}=5$ ms, $\tau_{r}=0.5$ ms, $\tau_{l}=1$ ms; The AMPA to NMDA ratio is 0.45^113^.

We simulated the network 500 times with a DC step current ($I_{DC}=4$) of duration 1.2 s. Synaptic noise was sampled from a normal distribution ($I_{syn-noise}\mathcal{\sim N}\left( \mu=0, \sigma=3 \right)$). We calculated the shuffled-corrected jittered cross-correlations between E and I population spike trains binned at 1ms within the 200ms time window (800-1000ms) after the initial transient response. Cross-correlations for different choices of $\sigma_{I}$ or $\sigma_{E}$ were reported as the average across columns (Figure S4C, S4E).

**Spike Train Cross-correlations.** The population cross-correlograms in Figure S4 report shuffled-corrected jittered cross-correlations^114,115^. We computed the jittered cross-correlations by resampling two spike trains within a specific time window such that for each spike in the original data, a spike is chosen at random with replacement from within the same time window, thus preserving the PSTH at the resolution of the jitter window. We computed the jittered cross-correlations with 4, 8, and 16 jitter windows, and the results of 8 jitter windows were shown for both the model and the experimental data. Shuffled cross-correlations were calculated by cross-correlating the first population spike train with the randomly permuted second population spike train. Both types of cross-correlations were averaged across trials and were further normalized by the geometric mean of the two spike trains' firing rates and a triangular function that corrects for overlap for the different lags. The normalized shuffled cross-correlation was then subtracted from the normalized jittered cross-correlation to produce the shuffled-corrected jittered cross-correlation.

We combined the spike trains of all model E neurons or all model I neurons within a column and computed the cross-correlation between the two population spike trains (Figure S4C, S4E). For the empirical data, we extracted neuronal spike trains during a 60-260ms time window after the stimulus and combined the spike trains of units from the same cluster to calculate their spike-time cross-correlations (Figure S4D, S4F). The reported results were averaged across trials from both attention conditions.

**Statistics.** We used the estimation statistics^48,116,117^ to display results of our main analyses such as the AMI of CRF parameters, the response latency, the CDI values, and the cross-correlations (Figure 1F, 2E, 2G, 3C, 3E, S2B, S2C, S2D, S3C, S4G, S5). Estimation statistics includes a swarm plot of all observed values and a bootstrap sampling distribution showing the magnitude of the effect and its likelihood. It avoids the pitfalls of significance testing such as false dichotomy by displaying the confidence of effect sizes instead of the p-values. In our study, we compared all visualized quantities (AMI, response latency, CDI, CCG) against 0 and generated their 95% bootstrap CIs via estimation statistics. If the CI of a distribution does not include 0, we have over 95% of confidence to claim that the mean value of the quantity is different from 0. If the CIs of two distributions do not overlap with each other, we have over 95% of confidence to claim that the mean values of the two distributions are different from each other. We also did non-parametric Kruskal-Wallis tests with Dunn and Sidák’s multiple comparison *post hoc* to verify our Bonferroni corrected Wilcoxon rank sum tests (Table S2). Our conclusions remain valid across statistical tests.

Table S2. ANOVA tests and Dunn and Sidák’s *post hoc*

| Figure Panel | ANOVA | Dunn and Sidák’s multiple comparison *post hoc* |
| --- | --- | --- |
| Figure 2E | Kruskal-Wallis:  **p = 4.085e^-4^** | p_narrow⇔medium_ = 0.896, **p_narrow⇔broad_ = 0.017**, **p_medium⇔broad_ = 7.228e^-4^** |
| Figure 3A | Kruskal-Wallis:  **p = 4.754e^-6^** | p_narrow⇔medium_ = 0.589, p_narrow⇔broad_ = 1.081e^-5^, **p_medium⇔broad_ = 0.001** |
| Figure 3C | Kruskal-Wallis:  **p = 0.006** | **p_narrow⇔medium_ = 0.031**, **p_narrow⇔broad_ = 0.007**, p_medium⇔broad_ = 0.995 |
| Figure 3D | Kruskal-Wallis:  **p_superficial_ = 0.001**, p_input_ = 0.103,  **p_deep_ = 0.003** | *Superficial*: p_narrow⇔medium_ = 0.994, **p_narrow⇔broad_ = 0.002**, **p_medium⇔broad_ = 0.013**;  *Input*: p_narrow⇔medium_ = 0.997, p_narrow⇔broad_ = 0.287, p_medium⇔broad_ = 0.186;  *Deep*: **p_narrow⇔medium_ = 0.028**, **p_narrow⇔broad_ = 0.004**, p_medium⇔broad_ = 0.984 |
| Figure 3E | Two-way ANOVA:  **p_cluster_ = 0.013**,  p_layer_ = 0.501,  p_interaction_ = 0.943 | *Cluster:* p_narrow⇔medium_ = 0.095, **p_narrow⇔broad_ = 0.013**, p_medium⇔broad_ = 0.946  *Layer:* p_super.⇔input_ = 0.994, p_super.⇔deep_ = 0.7823, p_input⇔deep_ = 0.588 |
| Figure S2A | Kruskal-Wallis:  p_all-layer_ = 0.175, p_superficial_ = 0.547, p_input_ = 0.568,  p_deep_ = 0.125 | *All layers*: p_narrow⇔medium_ = 0.794, p_narrow⇔broad_ = 0.180, p_medium⇔broad_ = 0.704;  *Superficial*: p_narrow⇔medium_ = 0.617, p_narrow⇔broad_ = 0.940, p_medium⇔broad_ = 0.923;  *Input*: p_narrow⇔medium_ = 0.822, p_narrow⇔broad_ = 0.999, p_medium⇔broad_ = 0.654;  *Deep*: p_narrow⇔medium_ = 0.805, p_narrow⇔broad_ = 0.132, p_medium⇔broad_ = 0.604 |
| Figure S2B | Kruskal-Wallis:  p_superficial_ = 0.405, p_input_ = 0.128,  **p_deep_ = 0.026** | *Superficial*: p_narrow⇔medium_ = 0.899, p_narrow⇔broad_ = 0.876, p_medium⇔broad_ = 0.450  *Input*: p_narrow⇔medium_ = 0.998, p_narrow⇔broad_ = 0.261, p_medium⇔broad_ = 0.282;  *Deep*: p_narrow⇔medium_ = 0.142, p_narrow⇔broad_ = 0.978, **p_medium⇔broad_ = 0.030** |
| Figure S2C | Kruskal-Wallis:  **p = 0.006** | p_narrow⇔medium_ = 0.298, p_narrow⇔broad_ = 0.439, **p_medium⇔broad_ = 0.004**; |
| Figure S5E | Kruskal-Wallis:  **p = 0.027** | p_narrow⇔medium_ = 0.999, p_narrow⇔broad_ = 0.208, p_medium⇔broad_ = 0.050 |
| Figure S5F | Kruskal-Wallis:  p = 0.106 | p_narrow⇔medium_ = 0.353, p_narrow⇔broad_ = 0.683, p_medium⇔broad_ = 0.155 |
| Figure S5I | Kruskal-Wallis:  p = 6.2e^-1^ | p_narrow⇔medium_ = 8.5e^-1^, p_narrow⇔broad_ = 7.0e^-1^, p_medium⇔broad_ = 9.9e^-1^ |
| Figure S5J | Kruskal-Wallis:  p = 6.1e^-2^ | p_narrow⇔medium_ = 9.3e^-1^, p_narrow⇔broad_ = 1.1e^-1^, p_medium⇔broad_ = 5.6e^-2^ |

**Supplementary Figure captions**

**Figure S1. Attention Effects on CRF Parameters for All Neurons**

(A) Schematics show the effect of positive attentional modulation of each parameter on the shape of CRF.

(B) Distributions of the squared norm of the residual from the CRF fitting of neurons in two attention conditions.

(C) Distributions of AMI of best-fitting parameters. The dashed lines mark the 0 modulation and the arrows indicate the median AMI values. The median AMI is significantly different from zero for each distribution (Wilcoxon signed rank test, *p* < 0.05).

**Figure S2. Cluster-wise Electrophysiological Properties**

See Table S2 in *SI Text* for ANOVA tests of (A), (B), (C).

(A) Mean firing rate ± SEM for visually responsive single units split by cell class or by layer. Neuronal firing rates were calculated from stimulus flashes with the highest common contrast across two monkey experiments in the "attend-away" condition. The number of single units within each cluster is shown. Clusters are not significantly different from each other in firing rate (Wilcoxon rank sum test, Bonferroni corrected, *n* = 3, *p* > 0.0167 for any pairs of comparisons across layers or within layers).

(B) Layer-wise distributions of mean response time for 3 classes (Narrow, N; Medium, M; Broad, B). The number of units is on top of each distribution. Dashed lines indicate the upper bounds of 95% CIs of Medium neurons. Distributions with CIs overlapping with the Medium class are shown in faded colors. Wilcoxon rank sum test reveals significant difference between Medium and Broad in the deep layer (Bonferroni corrected, *n* = 3, *p*_medium⇔broad_ < 0.0167).

(C) Firing rate estimates (mean ± SEM) and response time for 3 clusters using fixed kernel bandwidths optimized unit-by-unit (Narrow, *n* = 59; Medium, *n* = 66; Broad, *n* = 89 neurons). The inset shows the bootstrap sampling distribution of the mean response time for each cluster. The response time was defined as the first of five consecutive time bins after stimulus onset that show firing rates higher than the maximum of the pre-stimulus period. The dash line in the inset indicates the upper bounds of the 95% CI of the Medium class. The difference between Medium and Broad was substantiated by the Wilcoxon rank sum test (Bonferroni corrected, *n* = 3, *p*_medium⇔broad_ < 0.01).

(D) The average standard deviation of waveforms (summed over the time dimension) is shown (Mean ± SEM) for Narrow (N), Medium (M), or Broad (B) cluster. Statistically significant differences between clusters are indicated by asterisks (*p* < 0.05) and double asterisks (*p* < 0.001) according to the two-way ANOVA with Dunn and Sidák’s multiple comparison post hoc.

(E) The swarm plot of AMIs of best-fitting CRF parameters for each cell class. The lines to the right of each group show the mean and the standard deviations.

(F) Normalized contrast response function in “attend-in” (red) and “attend-away” (blue) conditions for every cluster in every layer. Mean ± SEM. We normalized each neuron’s CRF by first subtracting the average spontaneous activity and dividing the result by the peak response, both during AWAY condition.

**Figure S3. AMIs and CDIs for Each Cell Class**

(A) The AMI as a function of contrast for individual units within each cell class.

(B) Layer-wise AMI (mean ± SEM) for all units, Narrow units, and non-narrow units as a function of contrast (*left*) or averaged across contrast (*right*). Asterisk indicates either the distribution is significantly different from zero (Wilcoxon signed rank test, *p* < 0.01) or two distributions are significantly different (Wilcoxon rank sum test, *p* < 0.05).

(C) The swarm plot of CDIs within each layer, including three clusters, the whole population and non-narrow units (Medium + Broad). The bootstrap distributions of mean CDI for the whole population and non-narrow units are shown on the right. Distributions with CIs inclusive of 0 are illustrated in faded colors. Significantly positive distributions were also supported by the Wilcoxon signed rank test (*p* < 0.05).

**Figure S4. Normalization Model of Attention and Spiking Network Models**

(A) The structure of the normalization model of attention. The left panel shows a pair of orientated grating stimuli with identical contrasts, acting as input to the model. The central black dot indicates the fixation point. The dashed red circle indicates the receptive field of the model neuron centered on the grating stimulus. The stimulus drive shown in the middle panel is a collection of neural activity driven by the stimuli. Neurons are arranged based on their receptive field center (horizontal position) and orientation preference (vertical position). The values of the stimulus drive are shown by brightness. The top panel shows the attention field as a function of the receptive field center and the orientation preference. In this case, attention is guided to the right stimulus position and does not vary with orientation. Gray areas indicate values of 1, and white areas indicate values greater than 1. The suppressive drive at the bottom is calculated from the point-by-point product of the stimulus drive and the attention field and then pooled over space and orientation according to the suppressive field size. The stimulus drive is multiplied by the attention field and then divided by the suppressive field to generate the output firing rates of model neurons (right panel).

(B) i, CDIs for simulated neurons in the normalization model with different stimulus sizes and attention field sizes. In each panel, we vary the stimulation field size relative to the attention field size (x-axis), and the suppressive field size relative to the stimulation field size (y-axis). The pattern of CDI holds for a broad range of values of stimulus size (5, 10, 20) and attention field size (10, 20, 30). ii, CDIs for simulated neurons in the normalization model with different types of inputs. We changed the stimulus drive input to the normalization model to have either a nonlinear or an attention-modulated contrast response function. We tested both the response gain (10% increase in overall response) and the contrast gain (1% of increase in detected contrast) effects. For these simulations, the attention field size is 30 and the stimulus size is 5. The pattern of CDI holds for different types of inputs.

(C) Simulations of a conductance-based E-I network with different inhibitory receptive field size. *Left*: Schematic of the E-I networks probing the effects of different inhibitory receptive field sizes. 800 E and 200 I units were evenly distributed in 10 local E-I networks or “columns”. Neurons within columns are mutually coupled. We modeled inhibitory receptive field size as the standard deviations ($\sigma_{I}$) of E-I connections ($W_{ie}$) across columns. We varied the range of inhibitory receptive field ($\sigma_{I}$, various shades of green) while keeping other within-column connections static ($W_{ee}$, $W_{ii}$, $W_{ei}$). *Middle*: Raster plot showing the spiking activity for all units organized by their column IDs (blue, I; red, E) in response to a step input. The box depicts a 200 ms window used for computing spike-time correlations between E and I populations. *Right*: Cross-correlograms (mean ± SEM) between local E and I populations with different inhibitory receptive field sizes. Cross-correlations were averaged across 10 columns. A larger inhibitory receptive field reduces the cross-correlation between local E and I populations.

(D) Cross-correlograms (mean ± SEM) between Narrow and Broad classes in the superficial, input, and deep layers. Cross-correlations were averaged across sessions. The arrows mark time intervals during which cross-correlations were averaged and compared against the deep layer. Asterisks indicate that the mean difference of cross-correlation between layers in the corresponding interval has a 95% CI above 0. For the full estimation statistics, see Figure S4G.

(E) *Left*: *Changes* in stimulation field size (white box) can also lead to the variation of CDIs across layers. *Middle*: We tested this hypothesis in the E-I network by adjusting the standard deviation of between-column E-E connections ($\sigma_{E}$, various shades of orange) while keeping other connections the same ($W_{ee}$, $W_{ii}$, $W_{ie}$). *Right*: Cross-correlograms (mean ± SEM) between within-column E and I populations suggest that different E receptive field sizes have little impact on the spike-time correlations of local neural activity.

(F) Cross-correlograms (mean ± SEM) between Narrow and Medium classes in the superficial, input, and deep layers. Cross-correlations were averaged across sessions. Asterisks indicate that the mean difference of cross-correlation between layers in the corresponding interval has a 95% CI above 0.

(G) The Cumming estimation plot showing the cross-correlation per session between Medium and Narrow or between Broad and Narrow classes and the mean difference of cross-correlation between the superficial (*S*) and deep (*D*) layers or between the input (*I*) and deep layers. Cross-correlations were averaged within the time interval shown on top.

**Figure S5. Consistency of Results across Subjects.**

(A, C, E, G, I, K) Analyses of data from monkey A. (B, D, F, H, J, L) Analyses of data from monkey C.

(A, B) Estimation plots of AMIs of CRF parameters for all units within a subject. Same format as Figure 1F.

(C, D) Classification of single units from each subject based on waveform width. *Left*: Information metrics for different number of clusters with purple dotted lines marking the optimal number of clusters. Same format as Figure 2A. *Right*: Distribution of peak-to-trough duration for all units from a subject colored by their cluster identities from the 3-cluster result. Same format as Figure 2C.

(E, F) Cluster-wise response latency for each subject. Same format and cluster identities as Figure 2E. See Table S2 in *SI Text* for ANOVA tests.

(G, H) Cluster-wise bootstrap sampling distributions of AMIs of CRF parameters for each subject. Same format and cluster identities as Figure 2F. Significant difference from 0 detected by the estimation statistics were also substantiated by the Wilcoxon signed rank test (*p* < 0.05) except for r_max_ of Narrow from Monkey A.

(I, J) Estimation plots of CDIs combined across layers for each monkey. Same format and cluster identities as Figure 3C. Wilcoxon signed rank tests supported the positive distributions of Medium and Broad in monkey A (*p* < 0.001).

(K, L) Layer-wise estimation plots of CDIs for each subject. Same format and cluster identities as Figure 3E. Positive mean CDIs of Medium and Broad in the input layer from monkey A were also confirmed by the Wilcoxon signed rank test (p < 0.05).

**Figure S6. Clustering with Different Recording Techniques.**

(A) Classification of single units with laminar recordings (*n* = 337). *Left*: Information metrics (AIC, BIC) for different number of clusters (*k*) with their difference curves in red. The optimal number of clusters (detected by the kneedle algorithm) was marked by purple dotted line. *Right*: The histogram of peak-to-trough duration colored by their cluster identities when *k* = 3.

(B) Same as (A) for recordings with single tungsten electrodes (*n* = 73).
