# Supplementary material for: Laminar compartmentalization of attention modulation in area V4 aligns with the demands of visual processing hierarchy in the cortex
Source: Sci Rep. 2023 Nov 9;13:19558. doi: 10.1038/s41598-023-46722-8 (PMC10636153; doi:10.1038/s41598-023-46722-8)
Supplement: Supplementary file 2 — Supplementary Figures. [file 41598_2023_46722_MOESM2_ESM.pdf]

Figure S1 - Wang

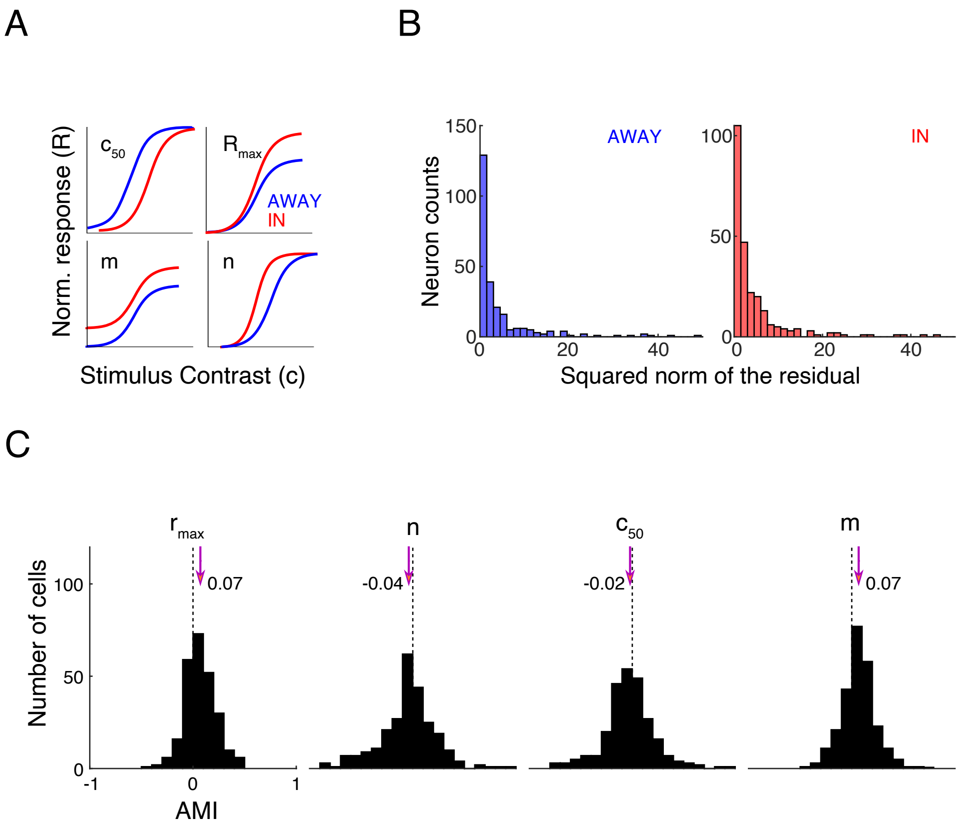

Figure S1. Attention Effects on CRF Parameters for All Neurons

(A) Schematics show the effect of positive attentional modulation of each parameter on the shape of CRF.

(B) Distributions of the squared norm of the residual from the CRF fitting of neurons in two attention conditions.

(C) Distributions of AMI of best-fitting parameters. The dashed lines mark the 0 modulation and the arrows indicate the median AI values. The median AMI is significantly different from zero for every distribution (Wilcoxon signed rank test, $p < 0.05$).

Figure S2 - Wang

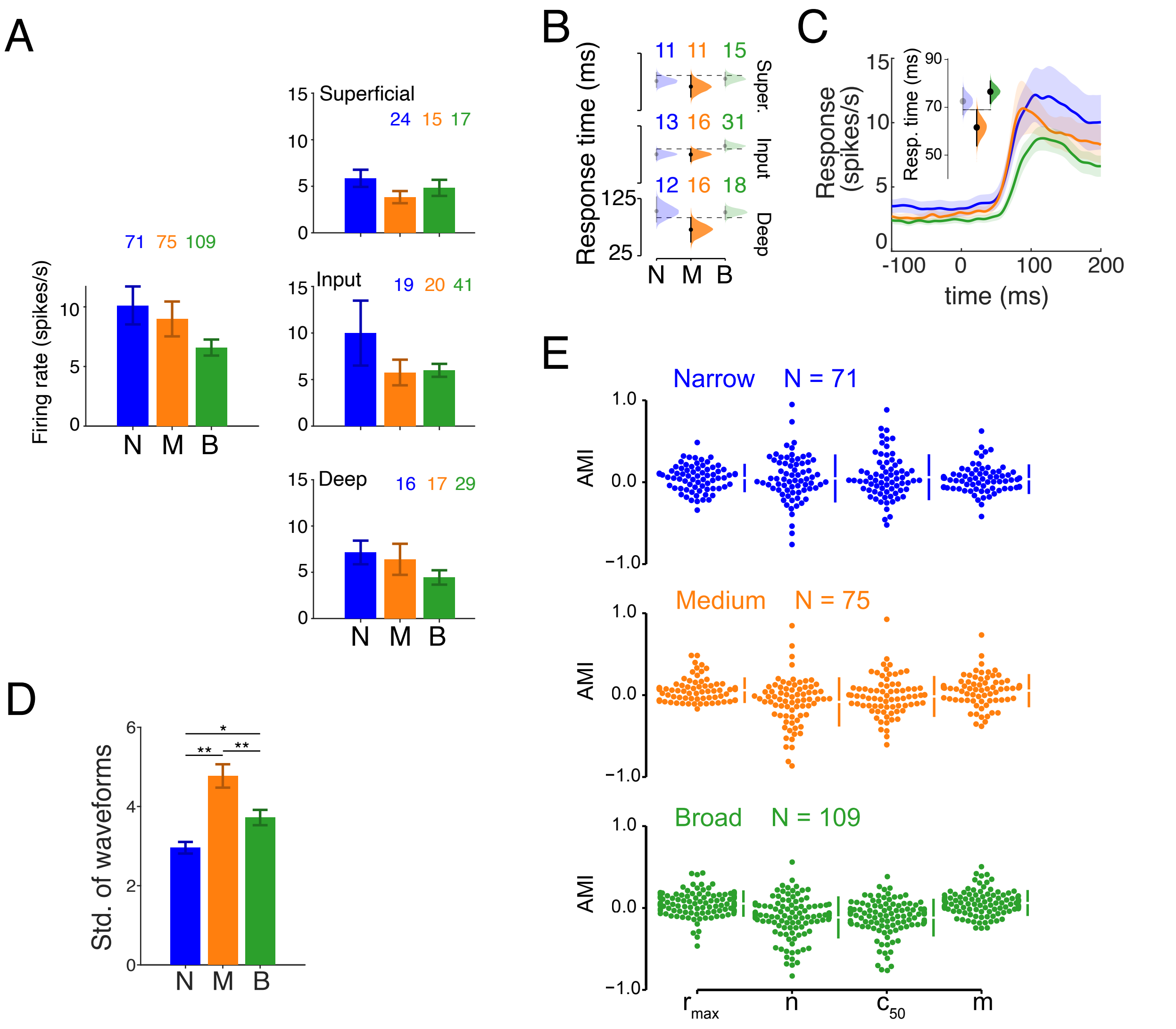

Figure S2. Cluster-wise Electrophysiological Properties

See Table S2 in *SI Text* for ANOVA tests of (A), (B), (C).

(A) Mean firing rate ± SEM for visually responsive single units split by cell class or by layer. Neuronal firing rates were calculated from stimulus flashes with the highest common contrast across two monkey experiments in the "attend-away" condition. The number of single units within each cluster is shown. Clusters are not significantly different from each other in firing rate (Wilcoxon rank sum test, Bonferroni corrected, $n = 3$, $p > 0.0167$ for any pairs of comparisons across layers or within layers).

(B) Layer-wise distributions of mean response time for 3 classes (Narrow, N; Medium, M; Broad, B). The number of units is on top of each distribution. Dashed lines indicate the upper bounds of 95% CIs of Medium neurons. Distributions with CIs overlapping with the Medium class are shown in faded colors. Wilcoxon rank sum test reveals significant difference between Medium and Broad in the deep layer (Bonferroni corrected, $n = 3$, $p_{medium<=>broad} < 0.0167$).

(C) Firing rate estimates (mean ± SEM) and response time for 3 clusters using fixed kernel bandwidths optimized unit-by-unit (Narrow, $n = 59$; Medium, $n = 66$; Broad, $n = 89$ neurons). The inset shows the bootstrap sampling distribution of the mean response time for each cluster. The response time was defined as the first of five consecutive time bins after stimulus onset that show firing rates higher than the maximum of the pre-stimulus period. The dash line in the inset indicates the upper bounds of the 95% CI of the Medium class. The difference between Medium and Broad was supported by the Wilcoxon rank sum test (Bonferroni corrected, $n = 3$, $p_{medium<=>broad} < 0.01$).

(D) The average standard deviation of waveforms (summed over the time dimension) is shown (Mean ± SEM) for Narrow (N), Medium (M), or Broad (B) cluster. Statistically significant differences between clusters are indicated by asterisks ($p < 0.05$) and double asterisks ($p < 0.001$) according to the two-way ANOVA with Dunn and Sidák's multiple comparison post hoc.

(E) The swarm plot of AMIs of best-fitting CRF parameters for each cell class. The lines to the right of each group show the mean and the standard deviations.

Figure S2 - Wang

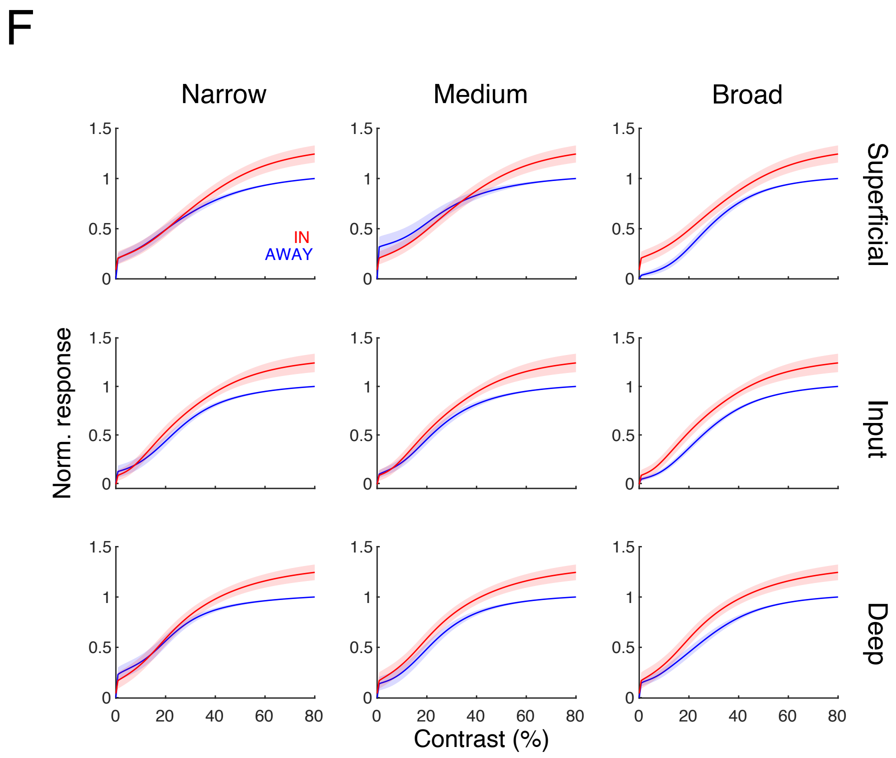

Figure S2. Cluster-wise Electrophysiological Properties
See Table S2 in *SI Text* for ANOVA tests of (A), (B), (C).
(F) Normalized contrast response function in “attend-in” (red) and “attend-away” (blue) conditions for every cluster in every layer. Mean ± SEM. We normalized each neuron’s CRF by first subtracting the average spontaneous activity and dividing the result by the peak response, both during AWAY condition.

Figure S3 - Wang

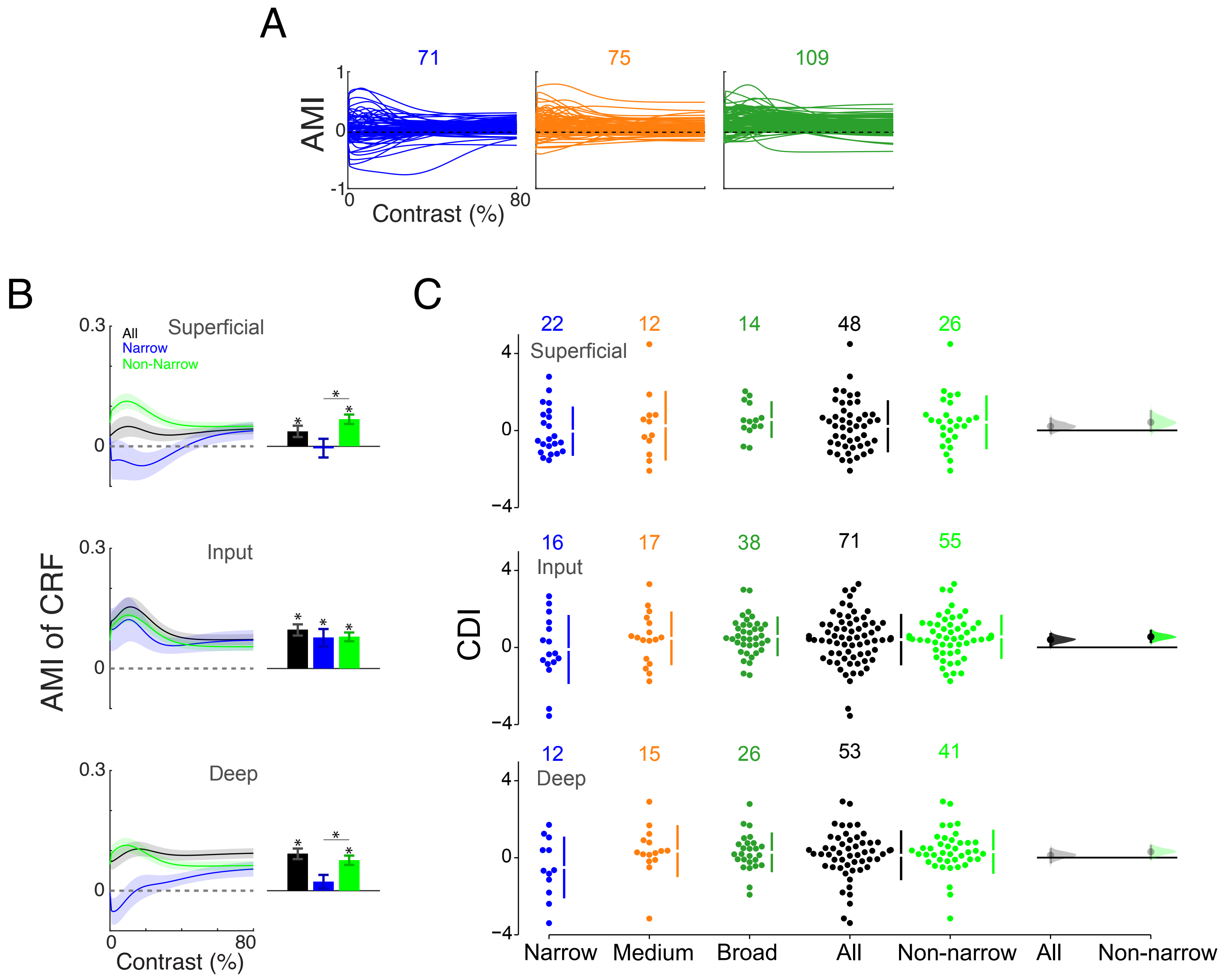

Figure S3. AMIs and CDIs for Different Cell Classes
(A) The AMI as a function of contrast for individual units within each cell class.
(B) Layer-wise AMI (mean ± SEM) for all units, Narrow units, and non-narrow units as a function of contrast (*left*) or averaged across contrast (*right*). Asterisk indicates either the distribution is significantly different from zero (Wilcoxon signed rank test, $p < 0.01$) or two distributions are significantly different (Wilcoxon rank sum test, $p < 0.05$).
(C) The swarm plot of CDIs within each layer, including three clusters, the whole population and non-narrow units (Medium + Broad). The bootstrap distributions of mean CDI for the whole population and non-narrow units are shown on the right. Distributions with CIs inclusive of 0 are illustrated in faded colors. Significantly positive distributions were also supported by the Wilcoxon signed rank test ($p < 0.05$).

Figure S4 - Wang

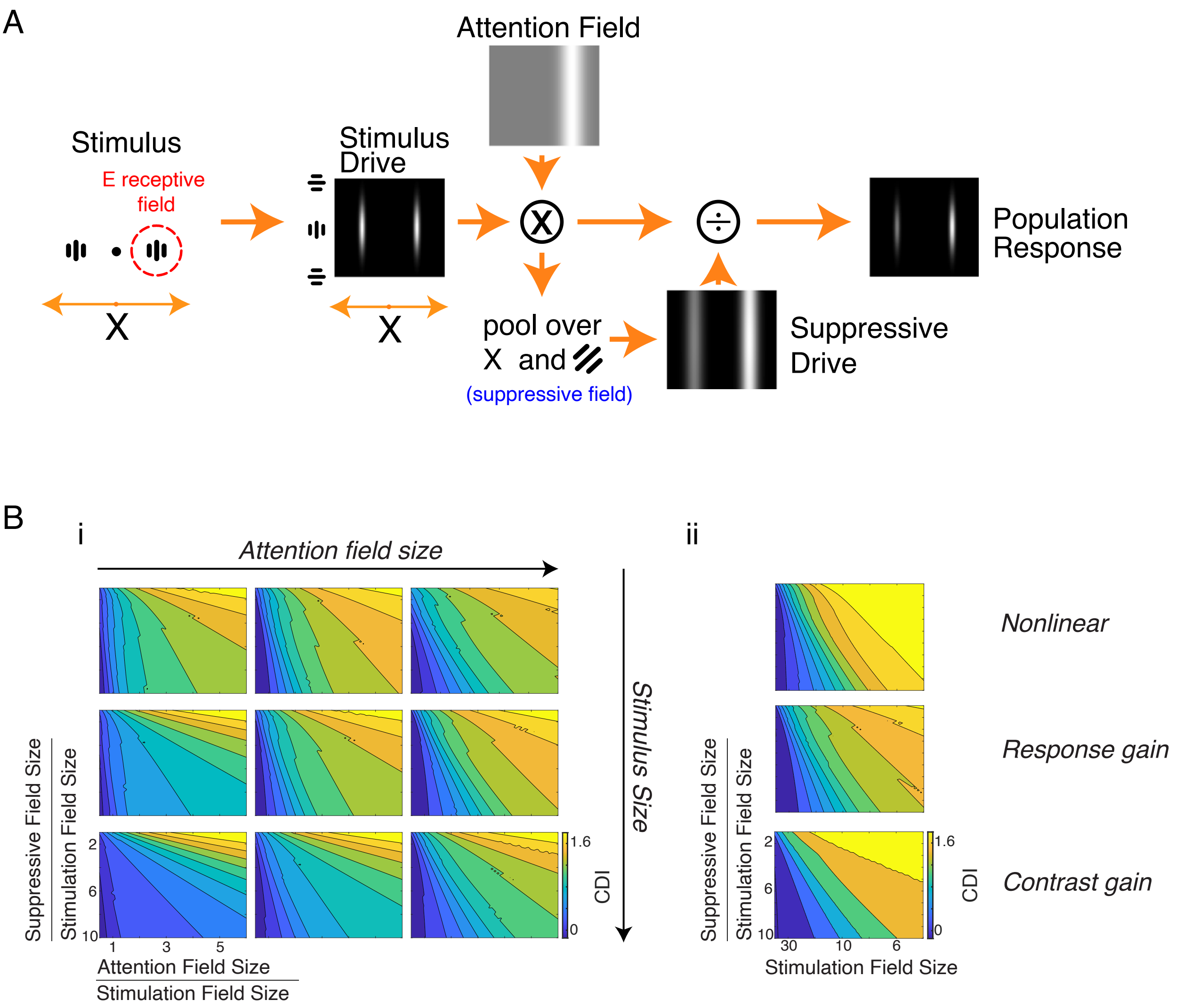

Figure S4. Normalization Model of Attention and Spiking Network Models

(A) The structure of the normalization model of attention. The left panel shows a pair of orientated grating stimuli with identical contrasts, acting as input to the model. The central black dot indicates the fixation point. The dashed red circle indicates the receptive field of the model neuron centered on the grating stimulus. The stimulus drive shown in the middle panel is a collection of neural activity driven by the stimuli. Neurons are arranged based on their receptive field center (horizontal position) and orientation preference (vertical position). The values of the stimulus drive are shown by brightness. The top panel shows the attention field as a function of the receptive field center and the orientation preference. In this case, attention is guided to the right stimulus position and does not vary with orientation. Gray areas indicate values of 1, and white areas indicate values greater than 1. The suppressive drive at the bottom is calculated from the point-by-point product of the stimulus drive and the attention field and then pooled over space and orientation according to the suppressive field size. The stimulus drive is multiplied by the attention field and then divided by the suppressive field to generate the output firing rates of model neurons (right panel).

(B) i, CDIs for simulated neurons in the normalization model with different stimulus sizes and attention field sizes. In each panel, we vary the stimulation field size relative to the attention field size (x-axis), and the suppressive field size relative to the stimulation field size (y-axis). The pattern of CDI holds for a broad range of values of stimulus size (5, 10, 20) and attention field size (10, 20, 30). ii, CDIs for simulated neurons in the normalization model with different types of inputs. We changed the stimulus drive input to the normalization model to have either a nonlinear or an attention-modulated contrast response function. We tested both the response gain (10% increase in overall response) and the contrast gain (1% of increase in detected contrast) effects. For these simulations, the attention field size is 30 and the stimulus size is 5. The pattern of CDI holds for different types of inputs.

Figure S4 - Wang

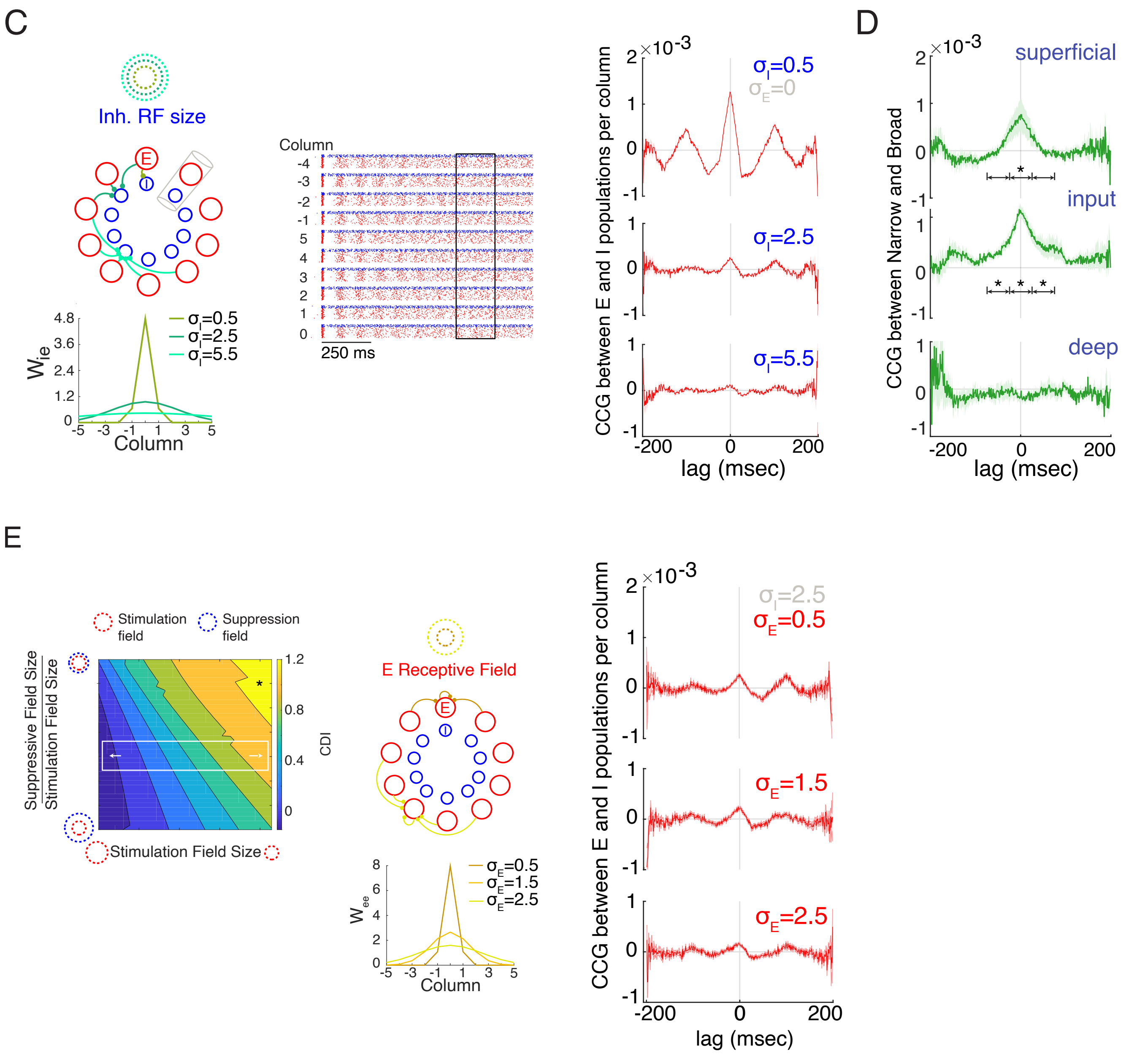

Figure S4. Normalization Model of Attention and Spiking Network Models

(C) Simulations of a conductance-based E-I network with different inhibitory receptive field size. *Left*: Schematic of the E-I networks probing the effects of different inhibitory receptive field sizes. 800 E and 200 I units were evenly distributed in 10 local E-I networks or "columns". Neurons within columns are mutually coupled. We modeled inhibitory receptive field size as the standard deviations (σ_I) of E-I connections (W_{ie}) across columns. We varied the range of inhibitory receptive field (σ_I, various shades of green) while keeping other within-column connections static (W_{ee}, W_{ii}, W_{ei}). *Middle*: Raster plot showing the spiking activity for all units organized by their column IDs (blue, I; red, E) in response to a step input. The box depicts a 200 ms window used for computing spike-time correlations between E and I populations. *Right*: Cross-correlograms (mean ± SEM) between local E and I populations with different inhibitory receptive field sizes. Cross-correlations were averaged across 10 columns. A larger inhibitory receptive field reduces the cross-correlation between local E and I populations.

(D) Cross-correlograms (mean ± SEM) between Narrow and Broad classes in the superficial, input, and deep layers. Cross-correlations were averaged across sessions. The arrows mark time intervals during which cross-correlations were averaged and compared against the deep layer. Asterisk indicates that the mean difference of cross-correlation between layers in the corresponding interval has a 95% CI above 0. For the full estimation statistics, see Figure S4G.

(E) *Left*: Changes in stimulation field size (white box) can also lead to the variation of CDIs across layers. *Middle*: We tested this hypothesis in the E-I network by adjusting the standard deviation of between-column E-E connections (σ_E, various shades of orange) while keeping other connections the same (W_{ee}, W_{ii}, W_{ie}). *Right*: Cross-correlograms (mean ± SEM) between within-column E and I populations suggest that different E receptive field sizes have little impact on the spike-time correlations of local neural activity.

Figure S4 - Wang

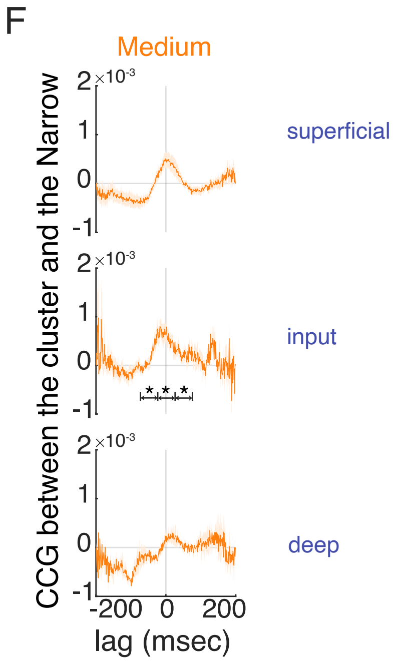

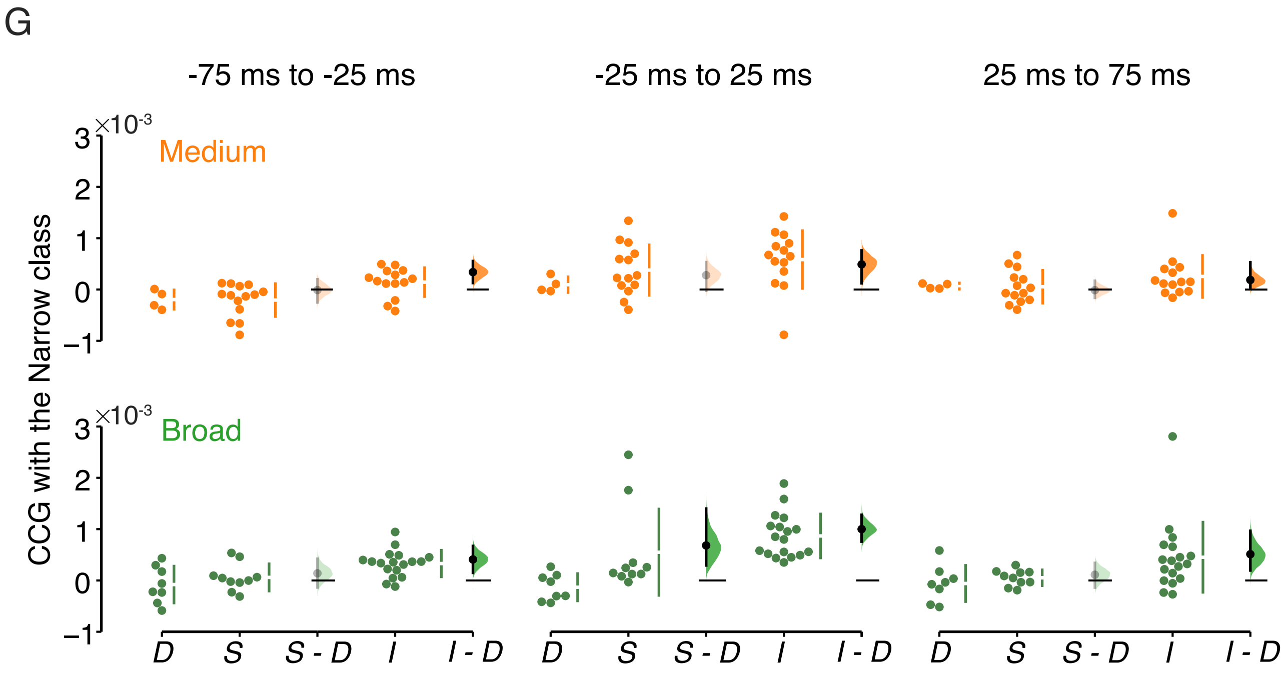

Figure S4. Normalization Model of Attention and Spiking Network Models

(F) Cross-correlograms (mean ± SEM) between Narrow and Medium classes in the superficial, input, and deep layers. Cross-correlations were averaged across sessions. Asterisk indicates that the mean difference of cross-correlation between layers in the corresponding interval has a 95% CI above 0.

(G) The Cumming estimation plot showing the cross-correlation per session between Medium and Narrow or between Broad and Narrow classes and the mean difference of cross-correlation between the superficial (*S*) and deep (*D*) layers or between the input (*I*) and deep layers. Cross-correlations were averaged within the time interval shown on top.

Figure S5 - Wang

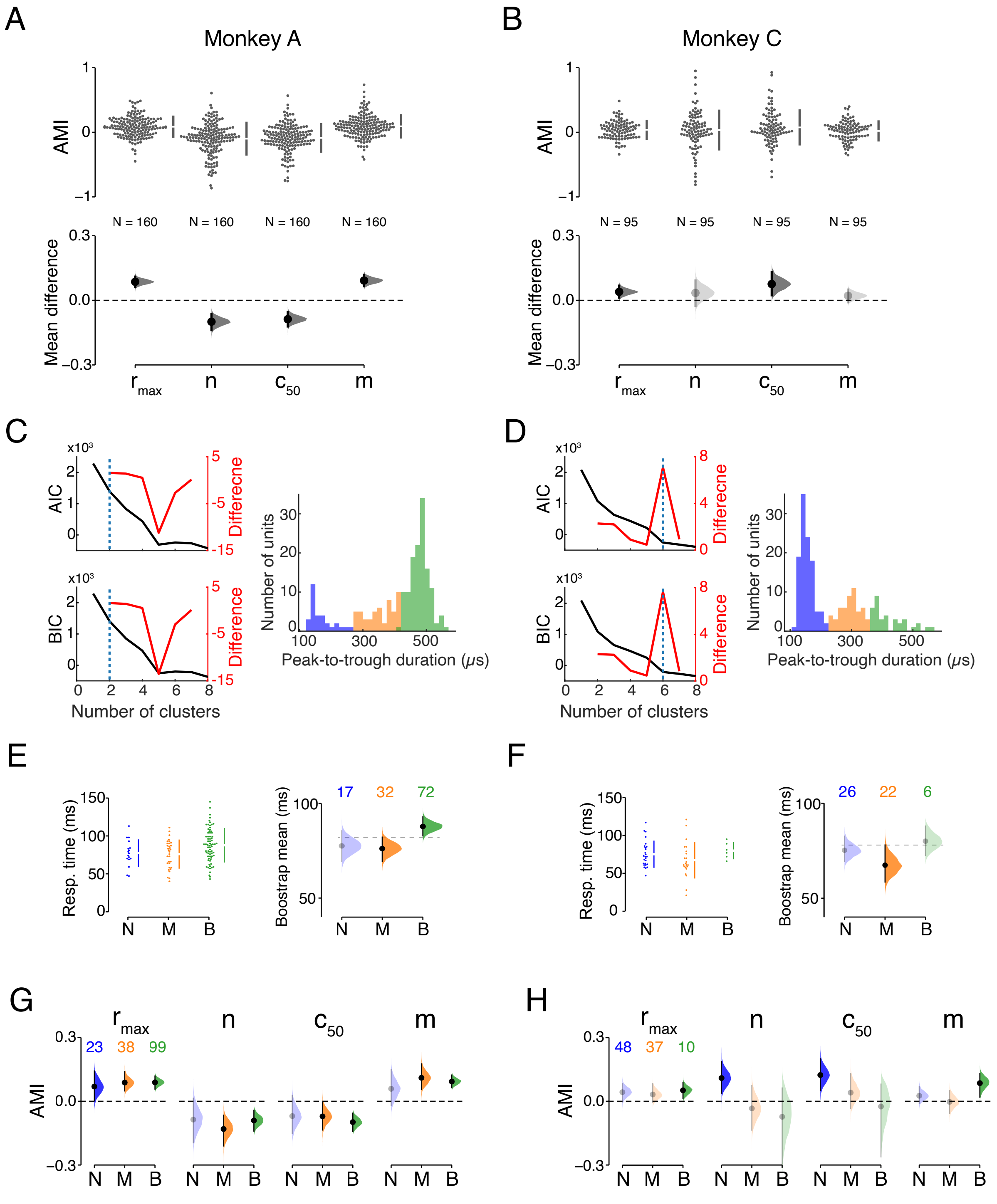

Figure S5. Consistency of Results across Subjects.
(A, C, E, G, I, K) Analyses of data from monkey A. (B, D, F, H, J, L) Analyses of data from monkey C.
(A, B) Estimation plots of AMIs of CRF parameters for all units within a subject. Same format as Figure 1E.
(C, D) Classification of single units from each subject based on waveform width. *Left*: Information metrics for different number of clusters with purple dotted lines marking the optimal number of clusters. Same format as Figure 2A. *Right*: Distribution of peak-to-trough duration for all units from a subject colored by their cluster identities from the 3-cluster result. Same format as Figure 2C.
(E, F) Cluster-wise response latency for each subject. Same format and cluster identities as Figure 2E. See Table S2 in *SI Text* for ANOVA tests.
(G, H) Cluster-wise bootstrap sampling distributions of AMIs of CRF parameters for each subject. Same format and cluster identities as Figure 2G. Significant difference from 0 detected by the estimation statistics were also substantiated by the Wilcoxon signed rank test ($p < 0.05$) except for r_{max} of Narrow from Monkey A.

Figure S5 - Wang

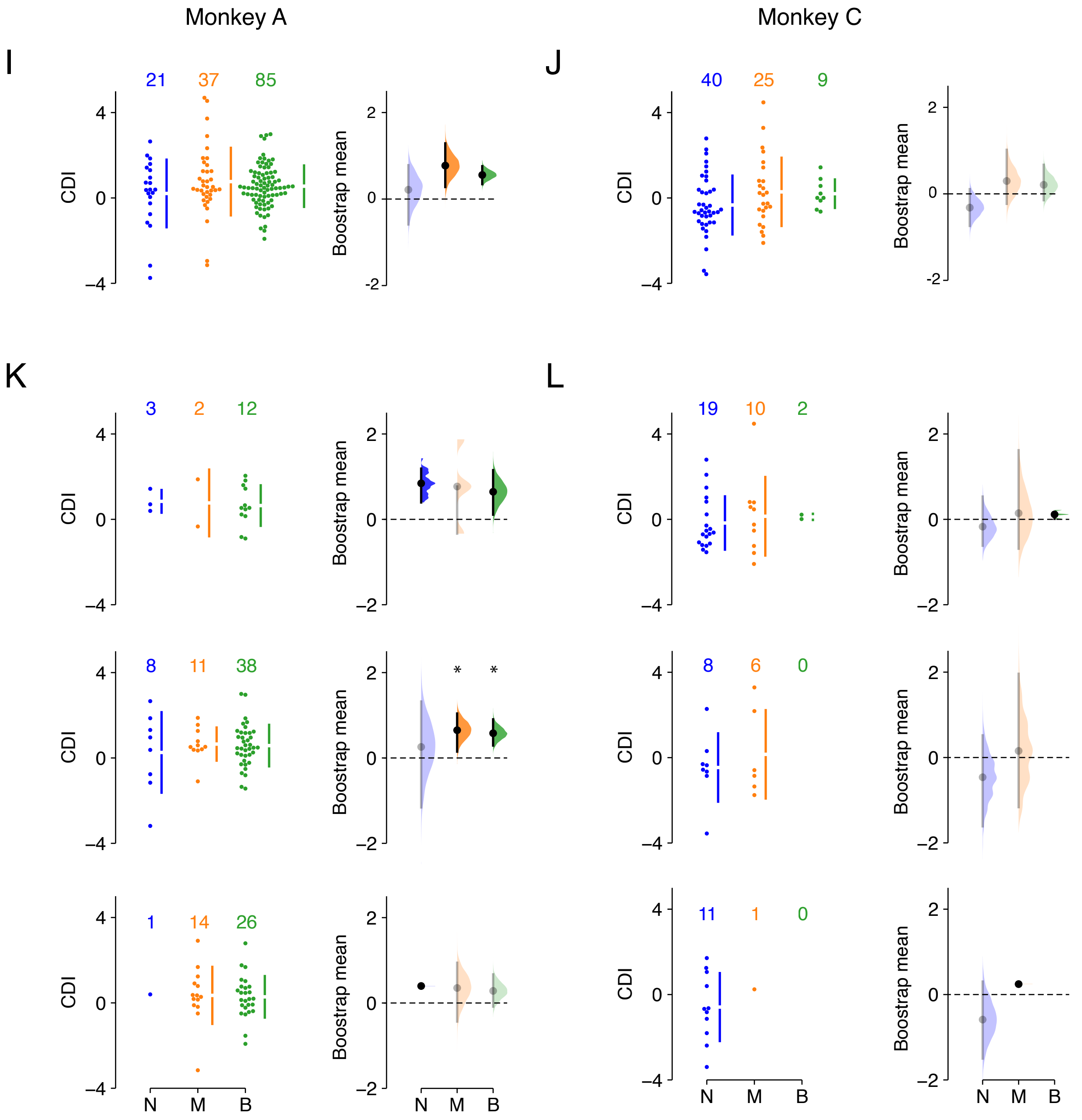

Figure S5. Consistency of Results across Subjects.
(A, C, E, G, I, K) Analyses of data from monkey A. (B, D, F, H, J, L) Analyses of data from monkey C.
(I, J) Estimation plots of CDIs combined across layers for each monkey. Same format and cluster identities as Figure 3C. Wilcoxon signed rank tests supported the positive distributions of Medium and Broad in monkey A ($p < 0.001$).
(K, L) Layer-wise estimation plots of CDIs for each subject. Same format and cluster identities as Figure 3E. Positive mean CDIs of Medium and Broad in the input layer from monkey A were also confirmed by the Wilcoxon signed rank test ($p < 0.05$).

Figure S6 - Wang

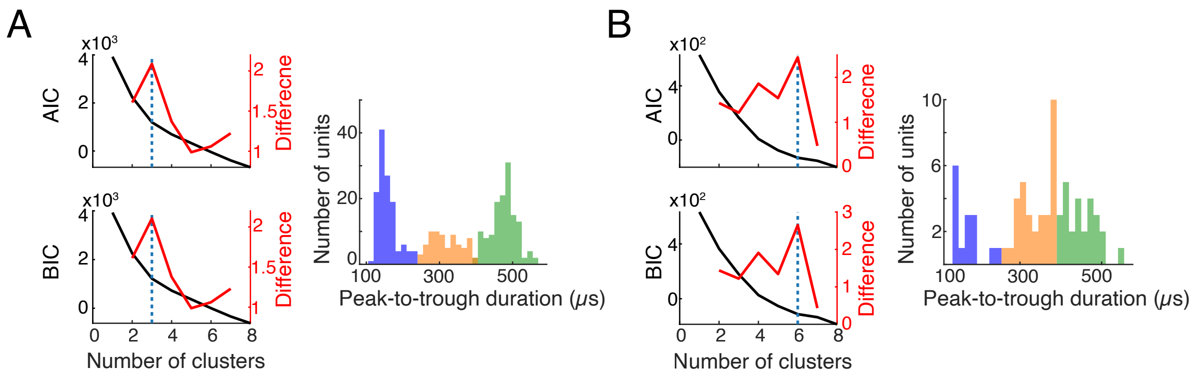

Figure S6. Clustering with Different Recording Techniques.
(A) Classification of single units with laminar recordings ($n = 337$). *Left*: Information metrics (AIC, BIC) for different number of clusters (k) with their difference curves in red. The optimal number of clusters (detected by the kneedle algorithm) was marked by purple dotted line. *Right*: The histogram of peak-to-trough duration colored by their cluster identities when $k = 3$.
(B) Same as (A) for recordings with tungsten electrodes ($n = 73$).
